# *Crataegus* Extract WS®1442 Stimulates Cardiomyogenesis and Angiogenesis From Stem Cells: A Possible New Pharmacology for Hawthorn?

**DOI:** 10.3389/fphar.2019.01357

**Published:** 2019-11-27

**Authors:** Jonas Halver, Kristin Wenzel, Jandirk Sendker, Carmen Carrillo García, Clemens A. J. Erdelmeier, Erik Willems, Mark Mercola, Nico Symma, Stephanie Könemann, Egon Koch, Andreas Hensel, Dennis Schade

**Affiliations:** ^1^Department of Chemistry and Chemical Biology, Technical University of Dortmund, Dortmund, Germany; ^2^Department of Internal Medicine B, University Medicine Greifswald, Greifswald, Germany; ^3^Partner site Greifswald, DZHK, German Centre for Cardiovascular Research, Greifswald, Germany; ^4^Institute of Pharmaceutical Biology and Phytochemistry, University of Münster, Münster, Germany; ^5^Department of Pharmaceutical and Medicinal Chemistry, Institute of Pharmacy, Christian-Albrechts-University of Kiel, Kiel, Germany; ^6^Preclinical Research, Dr. Willmar Schwabe GmbH & Co. KG, Karlsruhe, Germany; ^7^Muscle Development and Regeneration Program, Sanford Burnham Prebys Medical Discovery Institute (SBP), La Jolla, United States; ^8^Stanford Cardiovascular Institute and Department of Medicine, Stanford University, Stanford, United States; ^9^Partner site Kiel, DZHK, German Centre for Cardiovascular Research, Kiel, Germany

**Keywords:** *crataegus* spp., regenerative medicine, stem cells, angiogenesis, oligomeric proanthocyanidines, cardiomyogenic differentiation, bioassay-guided fractionation

## Abstract

Extracts from the leaves and flowers of *Crataegus* spp. (i.e., hawthorn species) have been traditionally used with documented preclinical and clinical activities in cardiovascular medicine. Based on reported positive effects on heart muscle after ischemic injury and the overall cardioprotective profile, the present study addressed potential contributions of *Crataegus* extracts to cardiopoietic differentiation from stem cells. The quantified *Crataegus* extract WS^®^1442 stimulated cardiomyogenesis from murine and human embryonic stem cells (ESCs). Mechanistically, this effect was found to be induced by promoting differentiation of cardiovascular progenitor cell populations but not by proliferation. Bioassay-guided fractionation, phytochemical and analytical profiling suggested high-molecular weight ingredients as the active principle with at least part of the activity due to oligomeric procyanidines (OPCs) with a degree of polymerization between 3 and 6 (DP3–6). Transcriptome profiling in mESCs suggested two main, plausible mechanisms: These were early, stress-associated cellular events along with the modulation of distinct developmental pathways, including the upregulation of brain-derived neurotrophic factor (BDNF) and retinoic acid as well as the inhibition of transforming growth factor β/bone morphogenetic protein (TGFβ/BMP) and fibroblast growth factor (FGF) signaling. In addition, WS^®^1442 stimulated angiogenesis *ex vivo* in Sca-1^+^ progenitor cells from adult mice hearts. These *in vitro* data provide evidence for a differentiation promoting activity of WS^®^1442 on distinct cardiovascular stem/progenitor cells that could be valuable for therapeutic heart regeneration after myocardial infarction. However, the *in vivo* relevance of this new pharmacological activity of *Crataegus* spp. remains to be investigated and active ingredients from bioactive fractions will have to be further characterized.

## Introduction

Natural products frequently serve as an inspiration and attractive starting point for the development of novel pharmacological agents (Newman and Cragg, 2012). In the present study, the aim was to investigate a complex plant-derived extract with documented use in cardiovascular medicine and which could be promising in the context of cardiac regeneration after myocardial infarction. Quantified extracts of the flowers and leaves of hawthorn (*Crataegus* spp.) have been used since decades for the adjuvant treatment of heart failure (i.e., NYHA I and II) (Koch and Malek, 2011; European Medicines Agency, 2016; European Pharmacopoeia, 2017). Based on this tradition and the documented safety they have been classified as “traditional herbal medicinal product” by the Committee for Herbal Medicinal Products of the European Medicines Agency (European Medicines Agency, 2016).

One of the most comprehensively studied hawthorn extracts is WS^®^1442 (Crataegutt^®^). Although no significant effect on mortality have been shown in a large clinical trial involving this extract (SPICE study, 2008) (Holubarsch et al., 2008), data from this and other *in vitro* and *in vivo* studies in humans and animals are indicating significant cardiovascular activity (Koch and Malek, 2011; European Pharmacopoeia, 2017). Besides efficacy in secondary endpoints, the large scale, long-term mortality trial did show that the use of WS^®^1442 is safe in patients receiving optimal medication for heart failure (Holubarsch et al., 2008). *Crataegus* extracts exhibit a pronounced pleiotropic pharmacological profile and, particularly regarding heart muscle physiology, several interesting activities have been reported: *Crataegus* extracts have a positive inotropic effect *via* a cAMP-independent mechanism. Protective effects within rat models of ischemic reperfusion after myocardial infarction have been described, which lead to a reduced spreading of the infarction area (Veveris et al., 2004). Such effects were mostly attributed to an unspecific anti-oxidant activity of oligomeric procyanidines (OPCs), but also specific signaling pathways involving the serine-threonine kinase Akt and the hypoxia-inducible factor 1 (HIF-1) have been suggested to play a role. In the context of cardiac hypertrophy, it has been shown that WS^®^1442 inhibits the phosphatase activity of calcineurin, an important trigger of cardiomyocyte growth (Koch and Spörl-Aich, 2006). Several other activities have been reported for hawthorn extracts, such as a decrease in the expression of atrial natriuretic factors (ANF) and fibronectin in rat models of hypertension and cardiac hypertrophy. Many mechanistic studies were performed in the context of vascular (patho)physiology since WS^®^1442 exhibits positive effects on the vascular endothelium. In this regard, an increased availability of nitric oxide (NO) has been shown along with the release of reactive oxygen species (ROS) which again trigger Src/PI3K/Akt signaling and inhibit PDGFβ-mediated signaling. In addition, vascular effects of WS^®^1442 were linked to the inhibition of Ca^2+^/PKC/RhoA-signaling and activation of cAMP/Rap1/Rac1 signaling (Furst et al., 2010; Bubik et al., 2011).

Based on the large number of positive effects on the myocardium after ischemic injury and the overall cardiovascular profile, we aimed at studying whether also mechanisms of cellular differentiation and regeneration could possibly play a role for hawthorn extract WS^®^1442. For this purpose, cardiac differentiation assays in murine and human embryonic stem cells as well as Sca-1^+^ progenitor cells isolated from murine hearts were used. Our results provide evidence for a differentiation promoting activity of WS^®^1442 that might be valuable for therapeutic heart regeneration after myocardial infarction.

## Materials and Methods

### Fractionation and Phytochemical Characterization of *Crataegus* Extract WS^®^1442

WS^®^1442 is a dry ethanolic (45% w/w, drug-to-solvent ratio 4–6.6:1) extract from hawthorn leaves with flowers according to the European Pharmacopoeia (European Medicines Agency, 2016), and was kindly provided by Dr. Willmar Schwabe GmbH & Co. KG. According to the European Pharmacopoeia, extracts from the hawthorn leaf and flower are derived from whole or cut, dried flower-bearing branches of *Crataegus monogyna* Jacq., *C. laevigata* (Poir.) DC. (syn. *C. oxyacanthoides* Thuill.; *C. oxyacantha* auct.) or their hybrids or, more rarely, other European *Crataegus* species including *C. pentagyna* Waldst. et Kit. ex Willd., *C. nigra* Waldst. et Kit., and *C. azarolus* L. Following the protocol by Vennat et al. (1992), 500 g of WS^®^1442 (Ch. 289 N001) were fractionated using Sephadex LH-20 column (22.5cm × 65cm; Pharmacia) chromatography by sequential elution with: 75 L demineralized water, 70 L ethanol 95% (v/v), 70 L methanol 100%, and 50 L acetone-water 70% (v/v). The volume of each individual eluent was collected in a single fraction to give the water fraction (= “water eluate,” yield 285 g), the ethanol fraction (= “EtOH eluate,” yield 59 g), the methanol fraction (= “MeOH eluate,” yield 68 g), and the acetone fraction (= “acetone eluate,” yield 29 g), respectively. According to a procedure described by Chatterjee et al. (1997), 400 g of WS^®^1442 (Ch. 289 N001) were partitioned twice between ethyl acetate and water. The combined ethyl acetate phases were concentrated and dried to give 42 g of fraction “ethylacetate.” From the remaining water phase, residual ethyl acetate was removed. Then ethanol was added to give a 40% ethanolic solution which was ultrafiltrated through a S1Y3 filter (Amicon, 3000 Dalton) (= “ultrafiltrate” fraction). The ultrafiltrate was concentrated and freeze-dried to give 264 g of fraction B. The retentate was washed off the filter using 60% (m/m) ethanol, then concentrated and freeze-dried to yield 75 g of the “ultraretentate” fraction.

Flavonoid quantification was performed according to the respective monograph of Ph.Eur. Ed. 9 (2017) and % flavones were calculated as hyperoside (European Medicines Agency, 2016). OPCs were determined with Folin-Ciocalteu reagent in a modified manner as described by Holzl and Strauch (1977), using (-)-epicatechin as standard (Holzl and Strauch, 1977).

### LC-MS- and LC-FD-Analytics

All samples (WS^®^1442 and fractions/eluates) were dissolved in acetonitrile-water (1:1, v/v), centrifuged, and then diluted to a final concentration of 10 mg/ml with the same solvent.

#### LC-MS methods

1) For the analysis of monomeric procyanidins, flavonoids, and other low molecular weight *Crataegus* ingredients, separation was performed on a Dionex Ultimate 3000 RS Liquid Chromatography System over a Dionex Acclaim PAII column (2.1 × 100 mm, 2.2 µm) with a binary gradient (A: water with 0.1% formic acid; B: acetonitrile with 0.1% formic acid) at 0.4 ml/min. 0 to 0.4 min: isocratic at 5% B; 0.4 to 9.9 min: linear from 5 to 100% B; 9.9 to 15.0 min: isocratic at 100% B; 15.0 to 15.1 min: linear from 100 to 5% B; 15.1 to 20.0 min: isocratic at 5% B. The injection volume was 2 µl. Eluted compounds were detected using a Dionex Ultimate DAD-3000 RS over a wavelength range of 200–400 nm and a Bruker Daltonics micrOTOF-QII time-of-flight mass spectrometer equipped with an Apollo electrospray ionization source in negative mode at 3 Hz over a mass range of *m/z* 50–1,500 using the following instrument settings: nebulizer gas nitrogen, 4 bar; dry gas nitrogen, 9 L/min, 200°C; capillary voltage 3,500 V; end plate offset -500 V; low mass 100 *m/z*; transfer time 100 µs, prepulse storage 6 µs; collision cell: 40 eV, isolation width 5, collision cell RF 130 Vpp. Internal dataset calibration (HPC mode) was performed for each analysis using the mass spectrum of a 10 mM solution of sodium formate in 50% isopropanol that was infused during LC reequilibration using a divert valve equipped with a 20 µl sample loop. 2) For the analysis of OPCs with DP > 1, the gradient was modified as follows: 0 to 0.4 min: isocratic at 10% B; 0.4 to 9.0 min: linear from 10 to 40% B; 9.0 to 9.1 min: linear from 40 to 100% B; 9.1 to 15.0 min: isocratic at 100% B; 15.0 to 15.1min: linear from 100 to 10% B; 15.1 to 20.0 min: isocratic at 10% B. The MS settings were modified as follows: mass range *m/z* 300–3,000; low mass 300 *m/z*; transfer time 140 µs; prepulse storage 15 µs, collision cell RF 600 Vpp. All other settings as stated above.

For OPCs, peaks were detected by base peak chromatograms of each deprotonated molecule’s most abundant isotopic signal (according to isotope pattern simulation) at its most abundant charge (empirical: DP1–4 z = 1, DP5–8 z = 2, DP > 8 z = 3) with a width of *m/z* ± 0.01. Peak heights were taken as signal size. Other compounds were detected using the Dissect Compounds algorithm of DataAnalysis 4.1 (Bruker Daltonics) and processed using an in-house VBA script. The signal intensity of each deprotonated molecule (z = 1) was used as signal size.

#### LC-FD method

Separation was performed on a Waters H-Class UHPLC system by an Acquity^®^ UPLC BEH200 SEC (1.7 µm, 4.6 × 150 mm) column with a binary gradient (A: acetonitrile: acetic acid [98: 2]; B: methanol: water: acetic acid [86: 12: 2]) at 1.0 ml/min. 0 to 6.18 min: linear from 0 to 35% B; 6.18 to 7.75 min: linear from 35 to 40% B; 7.75 to 9 min: isocratic at 40% B; 9 to 10.5 min: 40 to 100% B; 10.5 to 13 min: isocratic at 100% B; 13 to 15 min: 100 to 0% B; 15 to 18 min: isocratic at 0% B. The injection volume was 5 µl. Eluted compounds were detected with a fluorescence detector with *λ*(ex) 280 nm and *λ*(em) 316 nm.

### Cell Culture

#### Cardiac Differentiation of Embryonic Stem Cells (ESCs)

##### mESC culture, differentiation, and automated high content image analysis

CGR8 mESC (ECACC catalog 07032901) is a germ-line competent cell line that was established from the inner cell mass of a 3.5 day male pre-implantation mouse embryo (*Mus musculus*, strain 129). CGR8 and CGR8-*Myh6*-GFP mESCs were grown under feeder-free conditions as monolayers and maintained in DMEM (Gibco^®^) supplemented with 10% fetal bovine serum (FBS, PAN Biotech), 1% L-glutamine (Gibco^®^), 1% non-essential amino acids (Gibco^®^), 1% penicillin/streptomycin (Gibco^®^), 0.1 mM 2-mercaptoethanol (Gibco^®^), and 15 ng/ml murine LIF (Dortmund Protein Facility). Cells were cultured on 0.1% gelatin-coated 6-well plates (at 37°C, 5% CO_2_) and passaged after 2–3 days when confluency reached 60–70% to preserve the undifferentiated phenotype. A modified protocol was used for mESC cardiac differentiation (Schade et al., 2012; Willems et al., 2012). Briefly, cells were trypsinized and cultured in differentiation media (at 37°C, 5% CO_2_) on 0.1% gelatin-coated 384-well plates at a density of 500 cells per 50 µl differentiation media per well (= d0). The differentiation medium was composed of DMEM (Gibco^®^) supplemented with 10% FBS (PAN Biotech), 1% L-glutamine (Gibco^®^), 1% non-essential amino acids (Gibco^®^), 1% penicillin/streptomycin (Gibco^®^), and 0.1 mM 2-mercaptoethanol (Gibco^®^). At day 3 of differentiation (= d3), medium was added to 75 µl assay volume. Cells were treated with indicated compounds/extract/fractions or DMSO from day 4 to day 6 and incubated until day 11. During this time, the medium was replaced every 48–72 h. At day 11 of differentiation (= d11) cells were fixed in 4% paraformaldehyde and incubated with DAPI for nucleic acid staining. *Myh6*-GFP positive cells were imaged on a MicroXL System (Molecular Devices) and GFP levels from images were quantified with MetaXpress Software, using a multi-wavelength scoring algorithm (Molecular Devices).

##### hESC culture, differentiation and automated high content image analysis

A directed differentiation protocol and image-based quantification of cardiogenesis was performed in H9-hESCs carrying an MYH6-mCherry reporter as described previously (Willems et al., 2011; Lanier et al., 2012). H9 is a human (male) blastocyst-derived, pluripotent embryonic stem cell line provided by the Wicell Research Institute (Madison, USA). Crataegutt^®^ (liquid formulation) was prepared by dilution with PBS to the desired concentrations, followed by sterile filtration. After replating differentiated hESCs into 384-well plates on day 4 of differentiation, cells were treated from day 5–9 with the dilutions. Subsequent cell culture and image analysis was done according to the previously described protocols (Willems et al., 2011; Lanier et al., 2012).

##### Immunocytochemistry and confocal microscopy

On d11, paraformaldehyde-fixed and DAPI-stained cells were permeabilized with 0.25% Triton X-100 in PBS for 30 min at RT. After blocking with 10% FBS in PBS, cells were incubated with mouse cardiac Troponin T antibody (Thermo Scientific) or mouse cardiac myosin heavy chain antibody (eBioscience) at dilutions between 1:100–1:200. After incubation at 4°C overnight, cells were washed several times with PBS and then incubated with the corresponding secondary antibody (1:1000) for 4 h at RT. Images of PBS-washed cells were obtained using a Molecular Devices MicroXL System (10x) and Leica SP5 confocal microscope (20x).

##### Flow cytometry

Differentiated mESC cultures were trypsinized and dissociated into single cell suspensions pooling 16–48 wells of the same condition in 384-well-plates. After centrifugation, cells were fixed in 4% paraformaldehyde and incubated for 10 min at RT. Fixed cells were centrifuged and incubated with mouse anti-myosin heavy chain eFluor 660^®^ (eBioscience, 1:100 in 0.25% saponine/2% FBS in PBS) for 1 h at RT in the dark. Following washes with 0.25% saponine/2% FBS in PBS, cells were resuspended in 500 µl 0.25% saponine/2% FBS in PBS and analyzed on a BD LSRII Aria instrument. FlowJo software was used for data analysis.

##### Cell viability and proliferation assays

Cell viability was determined with the CytoScan^™^ WST-1 Cell Cytotoxicity Assay (G-Biosciences) according to the manufacturer’s instructions. Assay dye solution was directly added to the cells in the culture medium at a final concentration of 10%. After incubation for 4 h at 37°C, absorbance was measured using a microplate reader (Tecan Infinite M1000) at 420–480 nm. Following the general immunostaining protocol (see above), proliferation status of the cells was determined by image-based quantification (Image Xpress, Molecular Devices, see above) of total cell number (DAPI count) and the number of Ki67 positive cells using Ki67 antibody (Santa Cruz) at a dilution of 1:100.

#### Sca-1^+^ Progenitor Cells From Adult Murine Hearts

##### Isolation and culture of Sca-1+ cells

Wild type Friend leukemia virus strain B (FVB) mice of either sex at an age of six to eight weeks were used for the experiments. All animal experiments were performed in accordance with the Guide for the Care and Use of Laboratory Animals published by the U.S. NIH (NIH Publication no. 85-23, revised 1985). Experimental protocols were approved by the local animal care committee of Mecklenburg-Vorpommern (Landesamt für Landwirtschaft, Lebensmittelsicherheit und Fischerei Mecklenburg-Vorpommern). Sca-1^+^ cells were isolated from healthy mouse hearts using magnetic cell sorting according to an adapted version of previously published protocols (Samal et al., 2012). Mice were anesthetized with isoflurane and sacrificed *via* cervical dislocation. Extracted hearts were minced and incubated with DNase (0.01%) and Liberase (28 U/ml) (Roche Applied Science) in buffer containing 1 x Hank’s buffered salt solution (Invitrogen), 10 mM HEPES, 30 mM taurine, 4 mM NaHCO_3_, at pH 7.2 for 15 min at 37°C. During incubation the homogenates were mixed thoroughly every 5 min and fresh buffer was added. Then the mixture was filtered through a 70 µm strainer to remove cardiomyocytes and undigested tissues. Lineage positive cells representing mature hematopoietic cells like T cells (CD4^+^, CD8^+^); B cells (B220^+^), macrophages (Mac-1^+^), granulocytes (Gr-1^+^), and erythrocytes (Ter-119^+^) were depleted using the lineage cell depletion kit from Miltenyi Biotec *via* magnetic cell sorting (AutoMACSpro), according to the manufacturer’s instructions. Remaining lineage negative cells were re-suspended in 90 µl MACS running buffer (phosphate buffered saline, pH 7.2, 2 mM EDTA, 0.5% bovine serum albumin) per 10^7^ cells and incubated with 10 µl of Biotin-conjugated anti-Sca-1 antibody (BD Biosciences Pharmingen) as provided by the supplier for 10 min at 4°C. Afterwards 2 ml of running buffer was added to the cell suspension followed by centrifugation at 300 × *g* (4°C, 10 min). Cell pellets were resuspended in 80 µl running buffer followed by incubation with 20 µl of anti-biotin micro beads (Miltenyi Biotec) per 10^7^ cells for 15 min at 4°C. Cells were sorted again using magnetic cell sorting. The magnetically labeled fraction was considered as Sca-1^+^ cells and the remaining heterogeneous cell population as Sca-1^-^. Sca-1^+^ cells were seeded at a density of 32.000 cells per cm^2^ in 25 cm^2^ culture flasks and cultivated using Dulbecco’s Modified Medium and Nutrient Mixture F-12 (DMEM/F12 3:1, Invitrogen) supplemented with 20% FBS (Invitrogen) at 37°C in humid air with 5% CO_2_ for 2 days to ensure sedimentation of fibroblast-like cells. Suspension cells were used for further experiments.

##### Live cell proliferation assay

For proliferation experiments, Xcelligence technology and the RTCA DP Analyzer (ACEA, Biosciences) were used. This system continuously monitors cell proliferation by microelectronic cellular impedance detection (= cell index). To determine the proliferation rate an E-Plate (16 wells) containing micro fabricated gold electrode arrays was used. The E-plates were coated with 0.1% gelatin (Cell Biologics) for 24 h at 37°C. Sca-1^+^ cells (1x10^4^ cells) were seeded using 200 µl of media containing WS^®^1442 and fractions/eluates. 1) WS^®^1442 100 µg/ml, 50 µg/ml, and 25 µg/ml; 2) Ultraretentate 25 µg/ml, 10 µg/ml, and 5 µg/ml; and 3) MeOH eluate 25 µg/ml, 10 µg/ml, and 5 µg/ml. Medium with DMSO (final concentration 0.1%) was used as control. The proliferation rate was monitored up to 72 h using the RTCA DP Analyzer software. Each proliferation experiment was repeated at least four times using cells from a separate isolation employing two technical replicates.

##### Quantification of the Sca-1+ fraction by FACS

The percentage of Sca-1 expressing cells after 72 h treatment with media supplemented with different concentrations of WS^®^1442, Ultraretentate or the MeOH extract was determined using flow cytometry (BD LSR II Flow Cytometer, BD Bioscience). Cells were stained with an antibody against Sca-1 (anti-Sca-1 PE-Vio770, Miltenyi Biotec) for 10 min at 4°C, washed twice with PBS, and analyzed regarding the presence of surface marker Sca-1.

##### Tube formation assay for angiogenesis

Tube forming capacity of Sca-1^+^ cells in response to media supplemented with WS^®^1442 or the methanol fraction was assessed using a fibrin gel *in vitro* angiogenesis assay kit (Merck). A mixture of fibrinogen and thrombin was pipetted into each well of a 24-well plate and left to polymerize for 60 min at 37°C. Sca-1^+^ cells were seeded with 1 ml of test media into fibrin-coated wells at a density of 10^4^ cells per well. Medium with DMSO was used as control. Cells were allowed to grow for 72 h before digital images of 3–4 random microscopic fields were acquired using a Keyence microscope. Tubule length was calculated using ImageJ software (U. S. National Institutes of Health).

### Western Blots

Cells were washed with PBS and lysed with ice cold Cell Lysis Buffer (Cell Signaling) supplemented with protease (Roche) and phosphatase inhibitor cocktails (Sigma Aldrich). The bicinchoninic acid method was used for protein quantification using Pierce BCA Protein Assay Kit (Thermo Scientific) according to the manufacturer’s protocol. Lysates were applied to SDS-PAGE (12% SDS-tris glycine) and transferred to 45 µm PVDF membranes (Merck Millipore), which were blocked and stained using Odyssey^®^ Blocking Buffer (Li-Cor). Primary antibodies were incubated overnight at 4°C using a GFP antibody (AnaSpec) and anti-myosin heavy chain (eBioscience) at a dilution of 1:100. Anti-α tubulin (Sigma Aldrich) and anti-Gapdh (Cell Signaling) were used as loading controls (1:5,000). Following washes with TBST buffer, secondary antibodies were incubated for 2 h at RT using goat anti-mouse IRDye680 (Li-Cor Bioscience) and goat anti-rabbit IRDye800 (Li-Cor Bioscience) at 1:10,000 dilutions. Imaging was done with an Odyssey CLx Imaging System (Li-Cor).

### RT-PCR

RNA was isolated from differentiating mESCs (32 wells of a 384-well-plate pooled) at indicated times using TRIsure^™^ reagent (Bioline) and phenol/chloroform extraction method according to the manufacturer’s protocol. cDNA samples, synthesized from total RNA with qScript cDNA Synthesis Kit (Quanta Bioscience), were run on a LightCycler 480 (Roche) using Takyon^™^ SYBR MasterMix (Eurogentec). Relative expression levels and significance were determined using the ΔΔCt method. The primer sequences are as follows: *Acta2* (smooth muscle cells): forward primer ACTACTGCCGAGCGTGAG, reverse primer TGCTGTTATAGGTGGTTTCGTG; *Pecam* (endothelial cells): forward primer GAAGCCAACAGCCATTACGG, reverse primer GGAGCCTTCCGTTCTTAGGG; *Mesp1* (cardiac mesoderm): forward primer CCATCGTTCCTGTACGCAGA, reverse primer CTAGAAGAGCCAGCATGTCG; *Mef2c* (cardiac progenitors): forward primer AGATCCCGATGCAGACGATT, reverse primer AGACCGCCTGTGTTACCTG; *Tnnt2* (cardiomyocytes): forward primer CAGAGGAGGCCAACGTAGAAG, reverse primer TCGATCAGAGTCTGTAGCTCATT.

### Transcriptome Analyses

Cardiac differentiation assays in mESCs were performed as described above and cells treated on day 4 with vehicle control (DMSO, n = 2), WS^®^1442 (0.25 mg/ml, n = 3), or MeOH eluate (0.1 mg/ml, n = 3) for either 6 or 24 h before cells were collected for RNA extraction (= total of 16 samples). RNA extraction was performed by OakLabs. Briefly, quality control of RNA samples was accomplished with the 2100 Bioanalyzer (Agilent Technologies) using the RNA 6000 Pico Kit. For quantity control, a photometrical measurement with the Nanodrop 2000 spectrophotometer (Thermo Scientific) was performed. For labeling, the Low Input QuickAmp Labeling Kit (Agilent Technologies) was used to generate fluorescent cRNA. For 1^st^ strand synthesis, either oligo-dT primer or a random primer/oligo-dT primer mixture was used. After 2^nd^ synthesis, an *in vitro* transcription for synthesis of cRNA labeled with cyanine 3-CTP was performed. For hybridization, the Agilent Gene Expression Hybridization Kit (Agilent Technologies) was used according to the manufacturer’s instructions. Fluorescence signals on microarrays were detected by the SureScan Microarray Scanner (Agilent Technologies). Data of all samples were quantile normalized using the ranked mean quantiles according to Bolstad et al. (2003). For functional annotation Qiagen IPA software was used. Commonly regulated genes upon WS1442- and MeOH eluate-treatment were further evaluated and considered to be relevant when log2 (fold change) > +1 (up-regulation) or < -1 (down-regulation), compared to the DMSO control (*p* value < 0.05).

### Statistical Analyses

All data are shown as mean ± SEM for at least three independent biological replicates. Statistical analyses were performed using one-way ANOVA or Student’s t-test for pairwise comparisons. For transcriptome analyses (see *Transcriptome Analyses*), OakLabs performed normalization and statistics according to Bolstad et al. (2003).

## Results

### Crataegus Extract WS^®^1442 Promotes Cardiomyogenesis From Pluripotent Stem Cells

**Figure 1:**
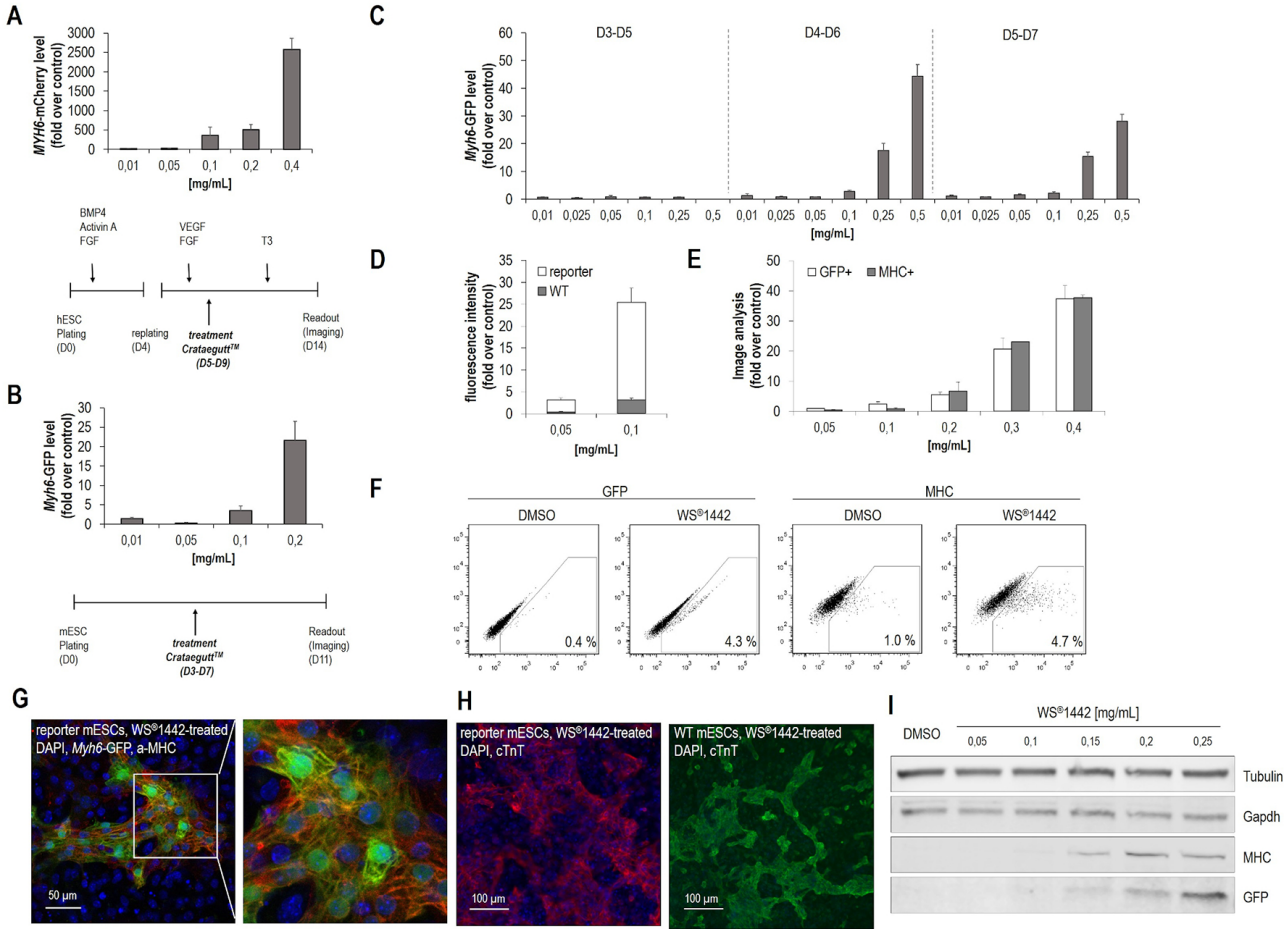
*Crataegus* extract WS^®^1442 stimulates cardiogenesis from human and murine embryonic stem cells. **(A**, **B)** Identification of WS^®^1442-induced cardiogenesis in **(A)** human and **(B)** murine embryonic stem cells after 5-day treatment with different concentrations of Crataegutt^®^. **(C)** Validation of dose- and time-dependent cardiogenic activity of WS^®^1442 in mESCs at different phases of differentiation. **(D)** Image-based quantification of unspecific fluorescence using mESCs from WT and reporter (*Myh6-*GFP) cell lines cultivated under the same conditions. **(E)** Correlation of αMHC- and αMHC-GFP expression of WS^®^1442-treated mESCs using anti-MHC-antibody. **(F)** Flow cytometry-based quantification of GFP^+^ and MHC^+^ cells after 2-day treatment of mESCs with 0.25 mg/ml WS^®^1442 or DMSO. **(G**, **H)** Immunostaining of day 11 mESCs from WT and reporter cell line to assess the expression of cardiomyocyte markers after 2-day treatment with 0.25 mg/ml WS^®^1442 from day 4 to day 6. **(I)** Validation of dose-dependent αMHC- and αMHC-GFP expression in WS^®^1442-treated mESCs (Western blot). WT = wild type.

Initial experiments were designed to detect cardiogenic effects of the finished dosage form, i.e. the proprietary medicinal product including its pharmaceutical adjuvants. For this, Crataegutt^®^ liquid was prepared under aseptic conditions for testing on cardiac differentiation from human (hESCs) and murine pluripotent embryonic stem cells (mESCs). A directed differentiation protocol was used on human embryonic stem cells (H9-hESCs, *MYH6*-mCherry reporter) (Willems et al., 2011). After mesoderm was formed, cells were replated on day 4 and treated with Crataegutt^®^ liquid at different concentrations during five days (**Figure 1A**). Cardiogenesis was stimulated in a concentration-dependent fashion. Moreover, a spontaneous differentiation protocol for murine embryonic stem cells (CGR8-mESCs, *Myh6*-eGFP reporter) was adapted and differentiating mESCs were exposed to Crataegutt^®^ liquid from day 3 to day 7 (**Figure 1B**) (Willems et al., 2012). Again, cardiomyocyte differentiation was effectively enhanced by Crataegutt^®^ with no signs of cell toxicity, but with the formation of functionally beating cardiomyocyte clusters that could be typically detected between day 10 to 11 of differentiation. Contributions of the adjuvants in the product (i.e., glycerol, propylene glycol, und sorbitol) to the observed activity could be excluded since these components were inactive in mESC cardiac differentiation (data not shown). Therefore, for all following studies WS^®^1442 extract was used, which revealed a similar dose-dependent activity as Crataegutt^®^ liquid. Cardiomyogenic activity was restricted to post-mesoderm phases of differentiation, with strongest effects during day 4–6 (**Figure 1C**).

To exclude potential unspecific fluorescence artefacts caused by either WS^®^1442 and/or *Myh6*-eGFP reporter cells, several validation experiments were performed (**Figure 1D–I**). Image-based analysis (in the GFP channel) was done with wild-type (WT) versus reporter mESCs both treated with WS^®^1442 between days 4–6 of differentiation (**Figure 1D**). Very low GFP fluorescence was captured in WT mESCs on day 11 indicating a detectable but low background fluorescence with the highest signal being attributed to *Myh6*-eGFP expression. Reporter signal correlation to endogenous expression of α-MHC was evaluated *via* side-by-side quantification of cardiomyocyte clusters. Simultaneous analysis of *Myh6*-eGFP (GFP-channel) and α-MHC (after immunostaining, Cy5 channel) revealed a high degree of correlation (**Figure 1E**). Confocal microscopy of formed *Myh6*-GFP^+^ and α-MHC-stained cardiomyocyte clusters are depicted in **Figure 1G**. **Video S1** illustrates beating cardiomyocyte clusters formed upon treatment with WS^®^1442. The correlation of GFP- and MHC-expression was further confirmed by Western blot analysis (**Figure 1I**). Absolute quantification of cardiogenesis was accomplished by flow cytometry on day 11 in WS^®^1442-treated reporter mESCs under spontaneous differentiation conditions (**Figure 1F**). WS^®^1442 treatment robustly induced cardiomyocyte formation up to a level of 4–5% (i.e., 5–10-fold over DMSO control). Moreover, WT CGR8-mESCs were differentiated under the same conditions as described for the CGR8-*Myh6*-eGFP reporter cells and exposed to WS^®^1442 between days 4–6. The cardiogenic effect could be visualized using a distinct, cardiomyocyte-specific marker (i.e., cTnT) which revealed similar phenotypes in WT and reporter cells (**Figure 1H**).

### Bioassay-Guided Fractionation and Phytochemical Analysis Reveal Distinct OPC Profiles and Suggest High-Molecular Weight Components as Bioactive Principle

**Figure 2:**
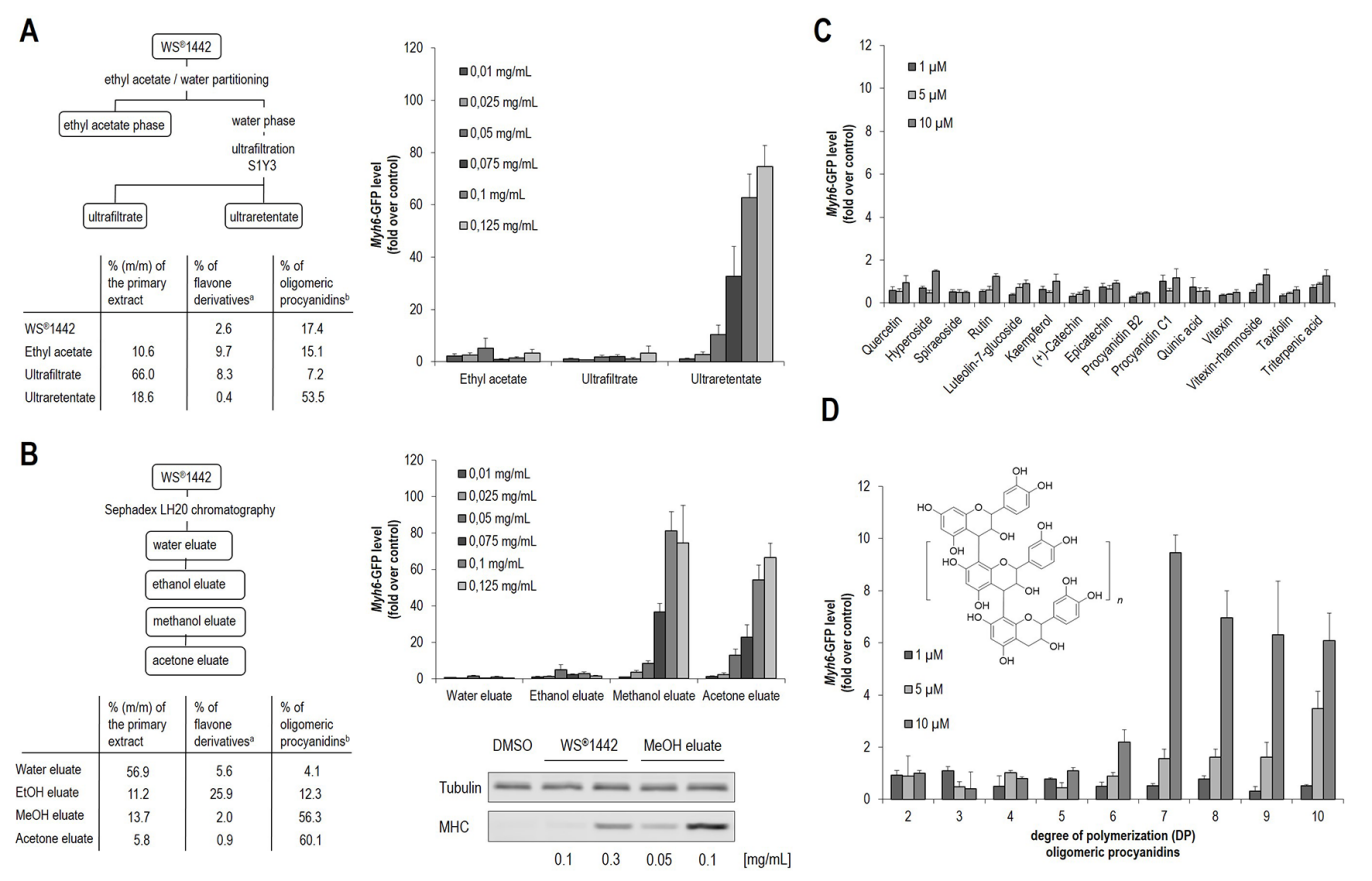
Evaluation of isolated WS^®^1442 fractions/eluates for cardiogenic activity in mESC differentiation. **(A)** Murine ES cells were cultured in 384-well format for 11 days and treated with different concentrations of fractions according to Chatterjee et al. from day 4 to day 6 (Chatterjee et al., 1997). **(B)** Concentration-dependent effect on cardiogenesis of mESCs after 2-day treatment with fractions/eluates using a protocol from Vennat et al. determined by Western blot and imaging analysis (Vennat et al., 1992). **(C**, **D)** Image-based quantification of cardiogenic activity after 2-day treatment with **(C)** *Crataegus*-related ingredients and **(D)** isolated oligomeric procyanidins (OPCs) with defined DP, where DP2 represents dimeric procyanidins, n = 1 equals DP3, n = 2 equals DP4, etc. ^a^ % Flavones quantified according to Ph. Eur. (Ed. 9), ^b^ % OPCs quantified with Folin-Ciocalteu reagent.

In order to gain insights into specific extract components that are responsible for the stimulation of cardiac differentiation, bioassay-guided fractionation of the extract WS^®^1442 was performed. Since WS^®^1442 has already been comprehensively characterized, the herein presented, initial approach was to use literature-described fractionation protocols to narrow down potential bioactive compounds or compound classes. Thus, WS^®^1442 was processed according to Chatterjee et al. (1997), yielding an ethyl acetate, ultrafiltrate and an ultraretentate fraction. All fractions were evaluated for potential cardiogenesis in differentiating mESCs (**Figure 2A**) (Chatterjee et al., 1997). Only the ultraretentate fraction exhibited a pronounced, concentration-dependent cardiogenic effect.

Since this fraction has been reported to contain mainly high-molecular weight components (Chatterjee et al., 1997), including oligomeric procyanidines (OPCs) (**Figure 2A**, 53.5%), a further fractionation protocol was employed according to Vennat et al. (1992) that enabled discrimination of OPCs with distinct degrees of polymerization (DPs) by preparative chromatography on a Sephadex^®^ LH20 stationary phase (Vennat et al., 1992). This protocol by Vennat et al. was originally described for the characterization of OPCs from *Hamamelis virginiana* L. and adapted to prepare comparable fractions from WS^®^1442 (**Figure 2B**). Fractionation was performed by a step gradient with water, ethanol (EtOH eluate), methanol (MeOH eluate), and acetone (acetone eluate). Phytochemical analysis of these eluates revealed a comparable composition as originally described for *Hamamelis* extract, with high concentrations of flavones in the EtOH eluate (25.9%) as well as high levels of OPCs in the MeOH (56.3%) and acetone (60.1%) eluates (see **Figure 2B**). The water eluate consisted of low molecular weight components and turned out to be inactive (**Figure 2B**). Also the DP1 OPC-enriched EtOH eluate, which contained mainly monomeric flavan-3-ols, dimeric and trimeric proanthocyanidins did not stimulate cardiac differentiation. However, the MeOH and acetone eluates showed strong concentration-dependent cardiogenic effects. **Video S2** shows beating cardiomyocyte clusters formed upon treatment with MeOH eluate. These results were in agreement with cardiogenic activity profiles of the fractions according to the protocol by Chatterjee et al. (1997), since high-molecular weight components appear to be responsible for the observed effects on cardiac differentiation.

In addition, a set of 15 single compounds was tested, which have been reported as *Crataegus* secondary metabolites. They were inactive in the cardiogenic assay (**Figure 2C**). Following the hypothesis that OPCs could account for the cardiogenic activity, defined OPC clusters were isolated from hawthorn as described previously, followed by systematic functional evaluation (Sendker et al., 2009; Zumdick et al., 2009). OPCs with an average DP of 7 or higher exhibited cardiogenic activity, although to a much lower extent (ca. 5–10-fold less effective) compared to WS^®^1442 extract, ultraretentate, and MeOH fractions, respectively.

Following the preliminary phytochemical analysis and functional testing of fractionated WS^®^1442 (**Figure 2A, B**), more elaborated analytical studies were needed to identify putative cardiogenic components. Thus, WS^®^1442 and its fractions/eluates were characterized by LC-MS using RP18 chromatography and by LC-FD using a diol stationary phase in HILIC mode. The latter method, separates OPCs according to their DP (Sendker et al., 2009; Zumdick et al., 2009). OPCs up to DP12 were detected in WS^®^1442 along with a polymer peak, which can be assumed to contain OPCs constituted of more than 12 flavan-3-ol units (see **Figure S2**).

**Figure 3:**
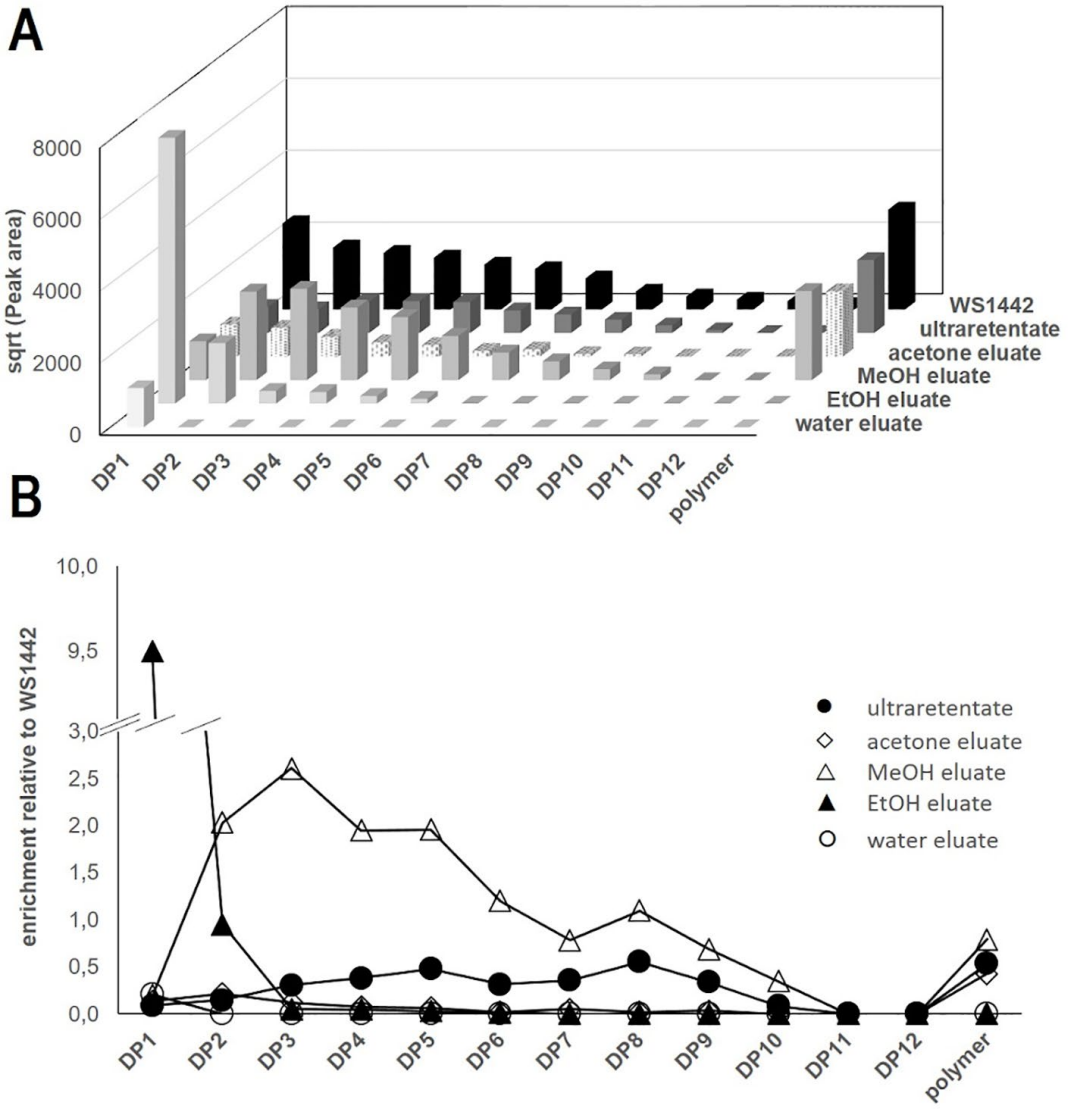
LC-FD-based characterization of WS^®^1442 and isolated fractions thereof. **(A)** Apparent distribution of oligomeric procyanidines (OPCs) and polymer peaks, data show the square root (sqrt) of the peak areas; **(B)** Enrichment of OPC and polymers relative to WS^®^1442 extract.

We found that the distribution of OPCs—based on DP—was rather homogenous in WS^®^1442 and gradually decreased with higher DP. In contrast, fractionation according to Vennat *et al*. (**Figure 2B**) yielded products of distinct OPC distribution (**Figure 3**): The water eluate was void of OPC apart of small amounts of flavan-3-ol monomers, which were enriched by almost 10-fold in the EtOH eluate. The major OPC part of DP2–10 eluted with MeOH with a particular strong enrichment of DP2–5. The OPC distribution of the acetone eluate resembled that of WS^®^1442 apart from smaller overall peak areas, obviously a consequence of the earlier elution with methanol.

The OPC distribution of the ultraretentate from the fractionation protocol by Chatterjee et al. (1997) showed an enrichment of DP4–9, although their absolute concentrations were about 50% lower than in WS^®^1442. Polymer peaks of comparable size were observable in all fractions except for the water and EtOH eluates.

In addition, LC-MS was used to analyze the composition of OPCs, OPC-related constituents, and other ingredients of WS^®^1442 and its fractions (**Table S1**). OPC patterns were observable by LC-MS up to DP8, whereas only diffuse increases in the extracted ion chromatograms indicated the presence of DP9–12 (**Figures S3**, **S4**). Cinchonains of DP1–6 were detected in WS^®^1442 and its fractions, with estimated relative amounts of 5–10% of their respective OPC for DP3–6 but 30–40% for DP1‒2 (**Table S1**). OPC hexosides (DP1–5) were characterized on the basis of their exact masses and fragmentation patterns (**Figures S4**, **S5**), with DP2 hexosides representing the most abundant ones (see **Table S1**). OPC hexosides up to DP2 have previously been described in *C. laevigata* and *C. monogyna* (Karar and Kuhnert, 2015). Low molecular weight compounds, e.g., flavonoids as monomers, caffeic acid derivatives, and triterpenes, were also detected using LC-MS and characterized by the product ion spectra of the deprotonated molecules (see **Tables S2**, **3**).

### WS^®^1442 and Its Active Fractions Stimulate Differentiation but not Proliferation of Cardiovascular Progenitor Cells *In Vitro*

**Figure 4:**
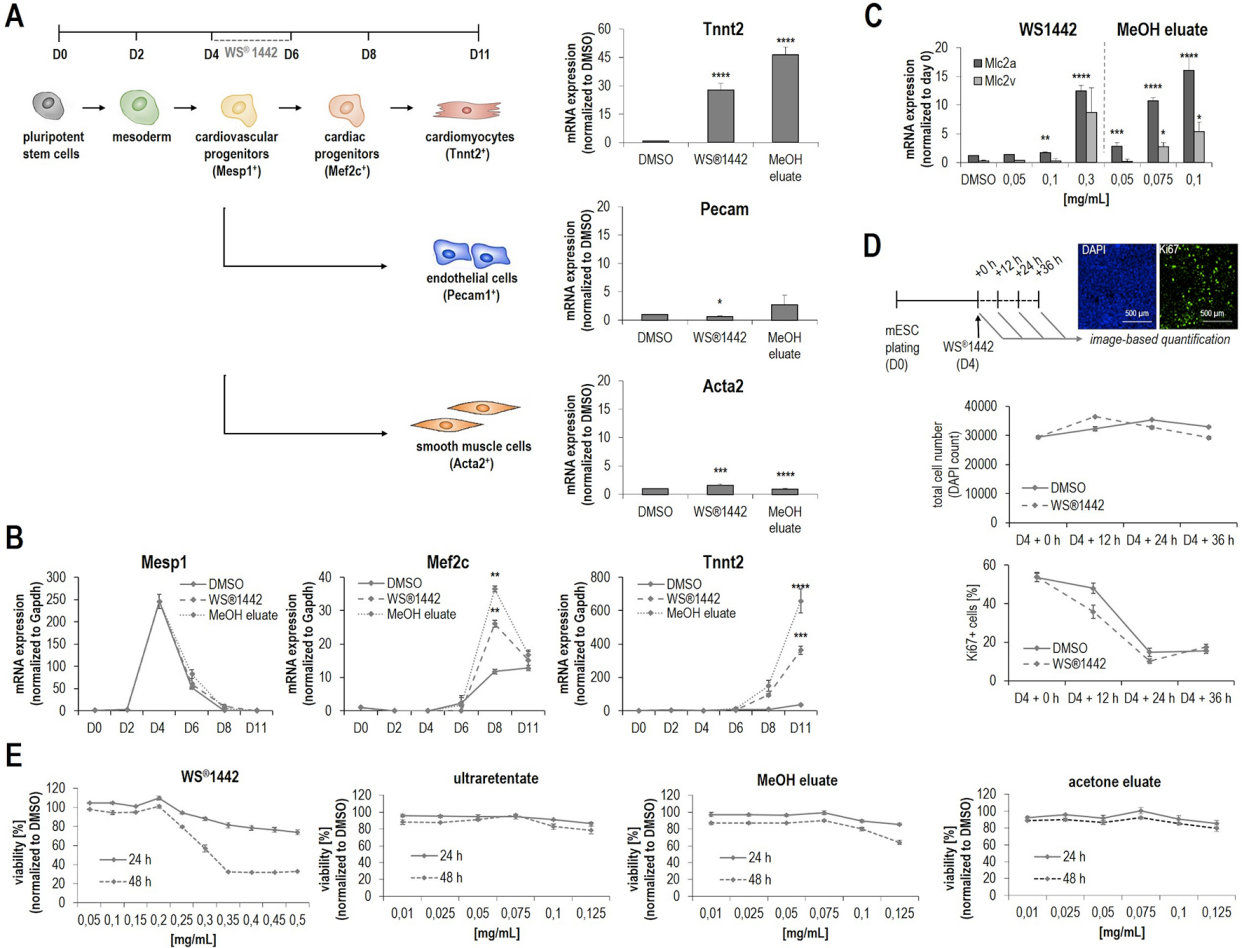
Characterization of WS^®^1442 and its active fractions on mesodermal differentiation cell-fates and cell proliferation during mESC differentiation. **(A**, **B)** Quantitative PCR-based measurement of **(A)** mesodermal and **(B)** cardiac differentiation markers of mESCs treated with DMSO, 0.25 mg/ml WS^®^1442 or 0.1 mg/ml MeOH eluate from day 4 to day 6. **(C)** Effect on cardiomyocyte subtype specification after 2-day treatment with WS^®^1442 and MeOH eluate (p ≤ 0.05 = *, p ≤ 0.01 = **, p ≤ 0.001 = ***, p ≤ 0.0001 = ****). **(D)** Image-based quantification of DAPI and Ki67 positive cells after 0-, 12-, 24-, and 36-h treatment of mESCs with 0.25 mg/ml WS^®^1442 or DMSO at day 4. **(E)** Cell proliferation and viability assay measuring metabolic activity on day 5 and day 6 mESCs after 1- and 2-day treatment with different doses of WS^®^1442 and fractions/eluates.

**Figure 5:**
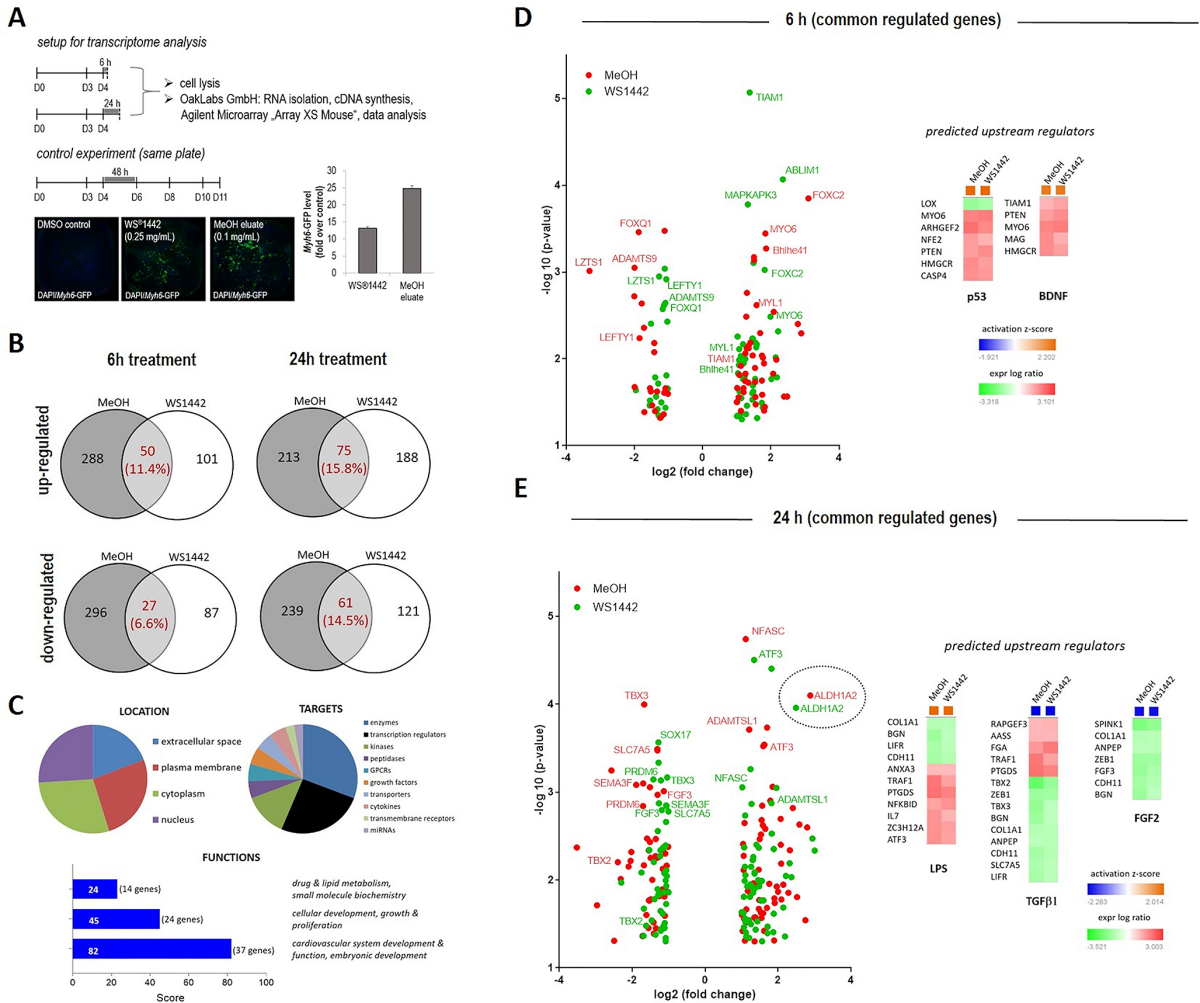
Transcriptome profiling of differentiating mESCs upon WS^®^1442 and MeOH eluate treatment. **(A)** Murine ES cells were cultured in a 384-format, treated with DMSO, 0.25 mg/ml WS^®^1442 or 0.1 mg/ml MeOH eluate at day 4. Part of the cells were collected after 6 h or 24 h for transcriptome analysis while the rest was cultured until day 11 (after 2-day treatment) for validation of cardiomyogenesis. **(B**–**E)** Annotation of commonly down- and up-regulated genes after **(B**, **D)** 6 h and **(B**, **E)** 24 h treatment with WS^®^1442 and MeOH eluate (log2[fold change] > 1 or -1, p-value < 0.05) using Qiagen IPS network analysis for **(C**–**E)** determination of upstream regulators, gene functions, targets, and location.

With analytically more defined, potently bioactive fractions in hand (i.e., ultraretentate and MeOH), WS^®^1442 was further characterized with regards to mesodermal and cardiovascular cell-fates in mESCs when applied between D4–6 of differentiation. **Figure 4A** shows the qPCR analysis of endothelial (*Pecam1*) and smooth muscle cell (*Acta2*) markers on day 11 of differentiation. The data revealed that the formation of vascular cells is significantly but very low stimulated by WS^®^1442 and MeOH eluate, whereas the cardiomyocyte marker *Tnnt2* is highly upregulated upon treatment (30- and 45-fold, respectively). Next, we asked whether certain mesodermal or cardiovascular progenitor cells are affected by WS^®^1442 or MeOH eluate treatment and determined the expression profiles of the mesodermal marker *Mesp1*, the cardiac progenitor cell marker *Mef2c*, and the cardiomyocyte marker *Tnnt2* by qPCR during the entire course of mESC differentiation (**Figure 4B**). Treatment with WS^®^1442 or MeOH did not alter *Mesp1* levels compared to DMSO while *Mef2c* was significantly upregulated. *Tnnt2* expression was also upregulated with a slight delay compared to *Mef2c* with highest levels on D11. Cardiomyocyte subtype-specific markers were analyzed on D11 of differentiation (**Figure 4C**). MeOH eluate-treated mESCs showed higher levels of the atrial marker *Mlc2a* compared to the ventricular marker *Mlc2v*. Next, we addressed whether these cardiovascular progenitor cells were indeed pushed towards cardiac differentiation or if WS^®^1442, MeOH eluate and ultraretentate promoted their proliferation. **Figure 4D** summarizes experiments in which WS^®^1442-treated mESCs were analyzed in a time-dependent fashion by automated imaging for cell number (nuclei, DAPI channel) and the cell cycle/proliferation marker Ki67 (GFP channel). Neither cell number nor cell cycle activity were significantly changed compared to DMSO treatment. Both DMSO- and WS^®^1442-treatment led to a decrease in Ki67-related proliferative activity in several independent experiments. This effect is most likely reflecting the continuous consumption of media supplements/serum with cell growth and differentiation over two days. However, we still asked whether cell viability (*via* quantification of reductive metabolism) is affected upon treatment. **Figure 4E** shows that WS^®^1442 and all its cardiogenic fractions (ultraretentate, MeOH, and acetone) do not disturb cell viability/metabolism after 24 h exposure. Only an extended treatment with WS^®^1442 and MeOH eluate had a negative effect on cell viability, a phenomenon that could be explained by stress-induced molecular mechanisms which were also suggested from transcriptome analyses (see *WS*^®^*1442 Stimulates Angiogenesis but Not Proliferation of Isolated Sca-1^+^* *Progenitor Cells From Murine Hearts Ex Vivo*, **Figure 5**).

### Genome-Wide Transcriptome Analysis Reveals Multiple Molecular Targets and Pathways

Changes in global gene expression patterns were addressed by transcriptome analysis of differentiating mESCs after treatment with WS^®^1442 (0.25 mg/ml) and of its active fraction MeOH eluate (0.1 mg/ml) between days 4–6. The experimental setup is outlined in **Figure 5A**: RNA was isolated for transcriptome profiling after 6 h and 24 h of treatment. To assure that mESC differentiation and the assay worked, controls were evaluated by image analysis from the same 384-well plate on day 11. These controls showed the typical 2-fold greater cardiogenic activity of the MeOH eluate compared to WS^®^1442. To confine the most relevant involved genes and signaling networks for later mechanistic interpretation, we focused on overlapping transcripts after WS^®^1442 and MeOH treatment. Only changes in gene expression with fold changes ≥2 and p-values of <0.05 were considered for data interpretation (see Volcano plots in **Figures 5D, E**).

The overall effect on gene expression was more moderate for WS^®^1442 compared to MeOH eluate. The data showed almost 2-fold more commonly regulated genes after 24 h compared to 6 h (18% versus 30%) after WS^®^1442- and MeOH treatment (**Figure 5B**). Gene ontology analysis and function annotation was performed using the Ingenuity Pathway Analysis (IPA) software algorithms and showed a prominent effect of WS^®^1442 and MeOH treatment on genes associated with cardiovascular development and function (score = 82, 37 genes) as well as cellular development, growth, and proliferation (score = 45, 24 genes). Mainly enzymes, kinases, and transcription factors were regulated. No specific subcellular localization of the molecular targets involved could be extrapolated from the transcriptome data since extracellular, plasma membrane, cytosolic, and nucleic targets appeared to be almost equally regulated.

IPA-based algorithms which allowed for a global analysis of the signaling networks and putative upstream regulators revealed that after 6 h particularly tumor suppressor p53 (p53) and brain-derived neurotrophic factor (BDNF) seem to be mechanistically involved. **Figure 5C** depicts these predicted upstream regulators along with their respective associated genes. The p53 (z-score +2.20) and BDNF (z-score +1.95) networks were predicted to be activated upon WS^®^1442 and MeOH eluate treatment. Several individual candidates of the commonly regulated genes from WS^®^1442 and MeOH eluate treatments are shown in Volcano plots (**Figures 5D, E**; a complete list is available in **Figure S6**), including downregulated effectors from the TGFβ superfamily of ligands (i.e., *Lefty1*, *Lzts1*, *Adamts9*) and members of the Fox transcription factors (e.g., *Foxc2*). While the transcriptome profiles at 6 h post-treatment provided insights into immediate cellular responses, global gene expression levels after 24 h should give information regarding the most dominantly affected factors and pathways that contribute to the cardiogenic phenotypes (**Figure 5E**). IPA analysis predicted several upstream regulators: In line with activated p53 after 6 h, many stress- and inflammation-associated genes were affected explaining the upregulation of a “lipopolysaccharide (LPS)” network of genes (z-score +2.01). On the other hand, TGFβ-1 (z-score -2.28) and FGF-2 (z-score -2.15) signaling networks were negatively regulated 24 h post-treatment. The Volcano plot in **Figure 5E** highlights *Aldh1a2* which was strongly upregulated (5–7-fold, p < 0.0001) both by WS^®^1442 and MeOH eluate implicating the involvement of retinoic acid signaling.

### WS^®^1442 Stimulates Angiogenesis But Not Proliferation of Isolated Sca-1^+^ Progenitor Cells From Murine Hearts *Ex Vivo*

**Figure 6:**
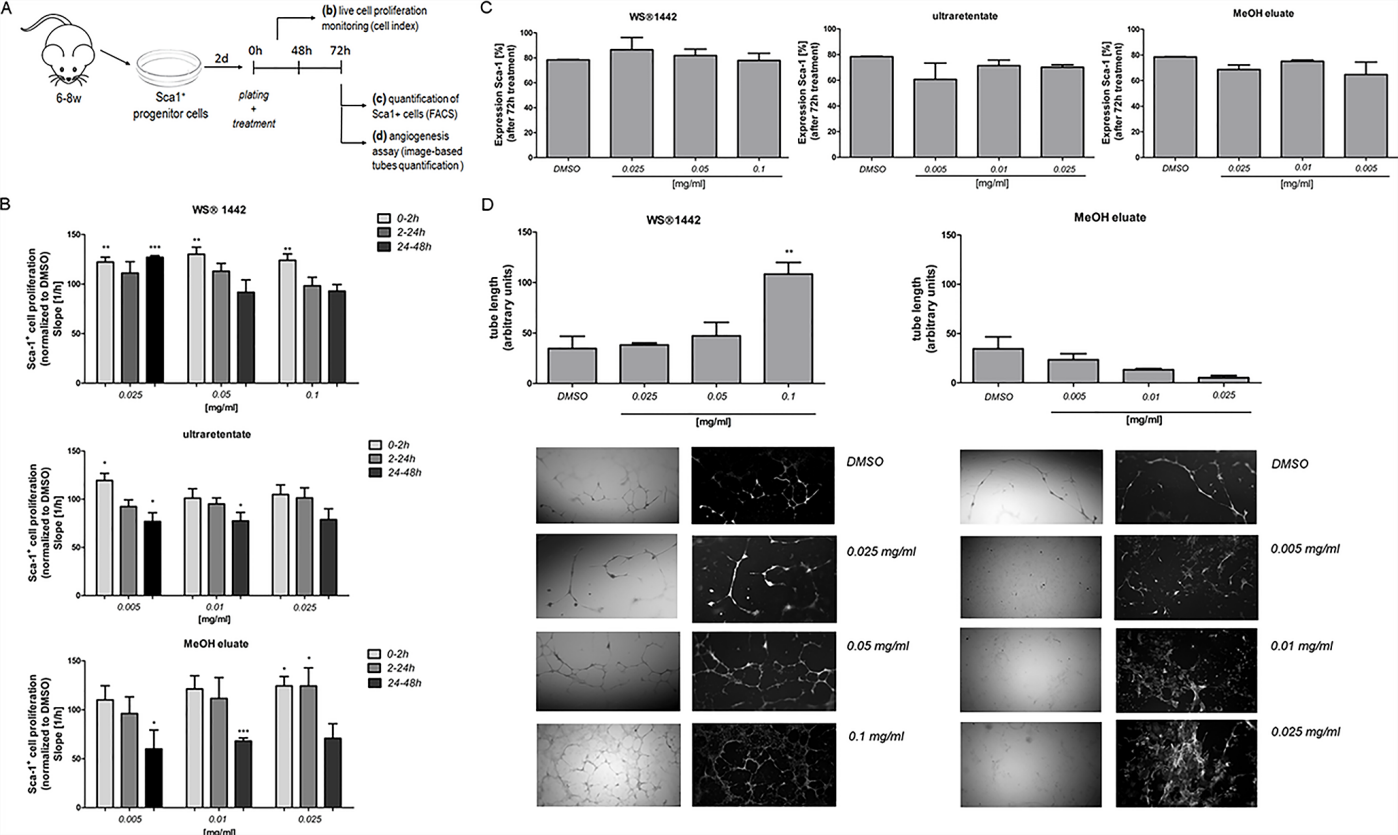
Evaluation of WS^®^1442 and its active fractions/eluates on proliferation and differentiation (angiogenesis) of primary murine Sca-1^+^ progenitor cells (Sca-PCs). **(A)** Isolation and culture of Sca-1^+^ cells for three distinct experimental setups; **(B)** Monitoring of Sca-PC attachment and proliferation by cell impedance measurements on 16-well plates, expressed as slope of the linear regression model with cell number as outcome and time as continuous independent variable (cell index, XCelligence RTCA DP Analyzer), Mean ± SEM, p ≤ 0.05 = *, p ≤ 0.01 = **, p ≤ 0.001 = *** vs. media plus DMSO (= 100%); **(C)** Quantification of Sca-1 expressing cells by flow cytometry (BD LSR II Flow Cytometer); **(D)** Tube formation assay using a fibrin gel *in vitro* angiogenesis assay kit, including image-based quantification of tube length (Mean ± SEM, p ≤ 0.01 = ** vs. media plus DMSO).

In an attempt to validate our findings in physiologically relevant progenitor cell populations within the heart, (van Vliet et al., 2012) Sca-1^+^ progenitor cells (Sca-PCs) were isolated from murine hearts (6–8-weeks-old mice) and treated with WS^®^1442, ultraretentate or MeOH eluate (**Figure 6A**) (Samal et al., 2012). WS^®^1442 and MeOH eluate facilitated and improved PCs’ attachment (**Figure 6B**, cell index after 2 h) but did not accelerate proliferation rates. Ultraretentate exhibited a more modest effect. In contrast to WS^®^1442, Sca-PC proliferation rates were slowed down after 24–48 h treatment with ultraretentate and MeOH eluate. Flow cytometry analysis of WS^®^1442- and MeOH eluate-treated progenitor cells after 72 h revealed no significant changes in the expansion of Sca-PC populations (**Figure 6C**).

We further evaluated the influence of WS^®^1442 on the tube forming ability of Sca-1^+^ cells for 72 h using an angiogenesis assay kit (**Figure 6D**). Quantitative analysis revealed a significant increase in tube length upon administration of WS^®^1442 100 µg/ml (2.2-fold, *p* < 0.01). Lower concentrations of WS^®^1442 did not cause a relevant change in tube formation of Sca-1^+^ cells compared to medium + DMSO (1.3-fold and 1.1-fold, respectively). On the other hand, treatment with the MeOH eluate kept Sca-1^+^ cells in a monolayer without marked tube formation. This finding indicates a concentration-dependent pro-angiogenic property of WS^®^1442 that can induce capillary-like tubule formation of Sca-1^+^ progenitor cells.

## Discussion

Hawthorn (*Crataegus* spp.) represents a traditional European medicinal plant. Extracts from leaves and flowers exhibit well-documented activities in cardiovascular medicine (Koch and Malek, 2011; European Pharmacopoeia, 2017), although the quantified hawthorn extract WS^®^1442 did not improve mortality in patients with congestive heart failure (SPICE study, 2008) (Holubarsch et al., 2008). Based on the long-standing tradition and the documented safety, the EMA classified preparations from *Crataegus* spp. leaves and flowers as “traditional herbal medicinal product” for “nervous cardiac complaints, mild mental stress and to aid sleep” in 2016 European Medicines Agency, 2016. Several studies suggest a positive effect of WS^®^1442 on heart muscle physiology (Koch and Malek, 2011; European Pharmacopoeia, 2017), including anti-hypertrophic activity as well as reduced scar sizes in a rat model of ischemic reperfusion (Veveris et al., 2004; Koch and Spörl-Aich, 2006). Hence, the aim of the present study was to explore principle mechanisms relevant for a regenerative potential of WS^®^1442 in patients suffering from heart failure after myocardial infarction.

Spontaneous and directed differentiation assays using pluripotent stem cells have been used as surrogate systems to probe cardiac differentiation and regeneration and dissect underlying mechanisms (Schade and Plowright, 2015; Spiering et al., 2015). In our previous work, we have harnessed such systems to identify small molecule stimulators of cardiomyogenesis to generate therapeutic modalities with regenerative potential for the infarcted heart (Willems et al., 2011; Lanier et al., 2012; Schade et al., 2012; Willems et al., 2012). Here, we showed that Crataegutt^®^ liquid, a finished dosage form of the standardized hawthorn extract WS^®^1442, efficiently stimulated cardiac differentiation both from human and murine ESCs in a concentration-dependent fashion (**Figures 1A, B**). This activity was further confirmed using WS^®^1442 dry extract with the most significant change between days 4–6 (**Figure 1C**), where typically cardiovascular progenitors have already been formed in this system (Willems et al., 2012; Cai et al., 2013). WS^®^1442-based stimulation of cardiac differentiation was in the same range (i.e., 30–50-fold over control) as that of optimized, single small molecules such as the TGFβ receptor type II degraders (Willems et al., 2012; Schade et al., 2012), leading to cardiomyocyte yields from mESCs in the 4–5% range (i.e., 5–10-fold over control). Unspecific fluorescence artefacts from this complex extract in the phenotypic, image-based readouts were ruled out in a series of experiments (**Figure 1D–I**).

To obtain first insights into specific compound classes responsible for the observed stimulation of cardiac differentiation, “bioassay-guided fractionations” were performed. The most abundant pharmacologically active substances of the leaves and flowers of hawthorn species are flavonoids (ranging between 1.5–2.5%) and catechin/epicatechin-derived oligomeric procyanidines (OPCs, up to 3.0%). Additional components belong to the classes of cinnamic acids, triterpenic acids (up to 0.6%), amines/catecholamines, and polymeric carbohydrates. (Hensel, 1998; Petereit and Nahrstedt, 2005) Quantified *Crataegus* extracts are well-defined in the European Pharmacopoeia (European Medicines Agency, 2016). The water/EtOH (45%) extract WS^®^1442 contains about 6.0% flavonoids and is standardized to 17.3‒20.1% OPC with a drug/extract ratio of 4‒6.6:1 (European Medicines Agency, 2016).

WS^®^1442 was fractionated according to a protocol described by Chatterjee et al. (1997) which partitions the extract between ethyl acetate and water, with the latter being subjected to ultrafiltration (**Figure 2A**) (Chatterjee et al., 1997). The resulting ethyl acetate, ultrafiltrate, and ultraretentate fractions were tested for cardiomyogenesis in mESCs. The ethyl acetate fraction has been reported to be enriched in low-molecular weight flavonoids (9.7%) and contains OPCs (15.1%), but was inactive. Similarly, the ultrafiltrate was inactive and contained flavonoids as well as possibly other water-soluble, small molecule components. Only the OPC-rich (53.5%) ultraretentate fraction dose-dependently stimulated cardiogenesis up to 80-fold over control (**Figure 2A**). This indicated that OPCs, or rather high-molecular weight components, were responsible for bioactivity. Hence, another fractionation procedure was employed that has been reported to separate OPCs dependent of their DP. Step-wise elution of WS^®^1442 on a Sephadex LH20 column resulted in water, EtOH, MeOH, and acetone eluate fractions (**Figure 2B**). Their composition in flavones and OPCs was in good agreement with those reported for *Hamamelis* (Vennat et al., 1992). Both OPC-rich fractions (i.e., MeOH and acetone eluates) were active in the mESC differentiation assay and stimulated cardiogenesis in a concentration-dependent fashion up to 70–80-fold (**Figure 2B**).

Our observation that fractions containing low-molecular weight components were inactive was further substantiated by screening 15 single substances from *Crataegus*. None of the tested flavone aglykons, flavone glucosides, catechin/epicatechin, dimeric and trimeric procyanidines, quinic and triterpenic acids stimulated cardiomyogenesis (**Figure 2C**). In an attempt to specifically address OPCs with defined DPs, single OPCs from DP2–10 were tested (**Figure 2D**) (Zumdick et al., 2009). None of the tested OPCs could achieve the same biological activity as the OPC-enriched ultraretentate, MeOH-, or acetone eluates. Only OPCs with a DP >7 showed low cardiogenic activity with moderate dose-dependency (see OPC DP10, **Figure 2D**).

One can speculate that the stability of such single OPCs might be compromised, and biological activities potentially require a mix of different OPCs. Therefore, all eluates from size exclusion chromatography as well as the OPC-enriched ultraretentate were comprehensively analyzed by LC-FD and LC-MS. Here, we disclose for the first time semi-quantitative OPC distribution profiles—along with their qualitative compositions—for WS^®^1442 and its fractions/eluates (**Figure 3**). OPCs up to DP12 could be detected using a diol stationary phase in HILIC mode (LC-FD). OPC quantities in WS^®^1442 gradually decreased with increasing DP (**Figure 3A**). The ultraretentate showed a similar OPC distribution profile as WS^®^1442 but lower absolute concentrations. OPC DP4–9 as well as the polymers’ peak were slightly enriched relative to WS^®^1442 (**Figure 3B**). The EtOH eluate has been reported to mainly constitute of monomeric flavan-3-ols and dimeric/trimeric OPCs (Vennat et al., 1992), which was in very good agreement with our OPC distribution (**Figure 3**). However, according to Vennat et al. (1992), the MeOH eluate was supposed to mainly contain DP4 OPCs and the acetone eluate OPCs with DP > 4 (Vennat et al., 1992). This could not be confirmed by our analyses as the MeOH eluate contained OPCs from DP2–10, with a clear enrichment of DP2–5 OPCs. The acetone eluate exhibited a similar OPC profile as WS^®^1442, but total OPC quantities were much smaller.

Together, these findings suggested that the cardiogenic activity of WS^®^1442 is not mediated by single small molecular compounds but is most likely attributable to larger molecules. When comparing the OPC distribution profiles of WS^®^1442 with those of all active fractions/eluates, DP3–5 OPCs are particularly enriched, and thus, represent a potentially pro-cardiogenic OPC cluster. This is interesting because an OPC-mediated differentiation-promoting effect has already been reported in the context of wound healing, although only for procyanidin B2 and not for DP3–4 OPCs (Kisseih et al., 2015). Another report described a potential regenerative activity of DP2–4 OPCs on dental mineralization *via* stimulation of dental pulp cells (Kulakowski et al., 2017). It should be noted that WS^®^1442 and all cardiogenic fractions/eluates contain significant amounts of, yet undefined, polymers. These polymeric components are enriched up to 1-fold in the bioactive ultraretentate and eluates. Thus, it is conceivable that at least part of the differentiation-promoting activity is conferred by highly polar, low-soluble polymers, such as polymeric carbohydrates. Future studies should specifically focus on OPC clusters DP3–6 and determine their constitutions. We already found unprecedented OPC *C*-hexosides in the present study (see *Supporting Information*). Additional preparative chromatography will be required in order to investigate the polymeric carbohydrate components. In general, it is not inconceivable that other herbal extracts with similar and ubiquitously present ingredients such as OPC clusters of DP3–6 exert comparable stimulating activities on cell differentiation.

The well-characterized ultraretentate and MeOH eluate with “enriched cardiogenic activities” as well as WS^®^1442 were further investigated with regards to mesodermal and cardiovascular cell-fates in differentiating mESCs. qPCR data for the vascular markers *Pecam1* (endothelium) and *Acta2* (smooth muscle) indicated that the formation of vascular cells is neither stimulated by WS^®^1442 nor by MeOH eluate, whereas *Tnnt2* (cardiomyocytes) was highly upregulated (30- and 45-fold) (**Figure 4A**). In addition, quantification of the atrial (*Mlc2a*) and ventricular (*Mlc2v*) cardiomyocyte markers pointed at a possible effect on cardiomyocyte subtype-specification (**Figure 4C**). In particular, MeOH eluate appeared to induce higher levels of the atrial marker *Mlc2a*. Directing subtype-specification during differentiation is of general interest in stem cell research as it improves the quality of available safety pharmacology models and screening platforms (Devalla et al., 2015; Cyganek et al., 2018). To date, several molecules and pathways have been described that favor distinct cardiac cell-fates. One prime factor for subtype specification *in vivo* is retinoic acid (RA) (Duester, 2008), which indeed seemed to be regulated by WS^®^1442 and MeOH eluate as transcriptome data suggested (see **Figure 5** and below).

We addressed if mesodermal or cardiovascular progenitor cells are affected by WS^®^1442 and MeOH eluate. qPCR-based expression analysis of *Mesp1* (mesoderm) and *Mef2c* (cardiac progenitor cells, CPCs) during the 11-day mESC differentiation assay revealed that mesodermal cells are not expanded but that transition to *Mef2c^+^* progenitor cells is stimulated (**Figure 4B**). With a slight delay compared to *Mef2c*, also *Tnnt2* expression was upregulated with highest levels on day 11 of differentiation. To address whether progenitor cells were indeed pushed towards cardiac cell-fates instead of being expanded by proliferation, WS^®^1442-, MeOH eluate-, and ultraretentate-treated (D4–6) mESC cultures were analyzed by automated imaging for cell number and the cell cycle/proliferation marker Ki67 (**Figure 4D**). However, no significant changes in cell number or cell cycle activity were detected. Together, these data underlined that WS^®^1442 and its bioactive fractions stimulated cardiovascular progenitor cells towards cardiac differentiation after mesodermal cells have been formed, whereas pro-proliferative effects did not contribute to cardiomyogenesis.

To gain first mechanistic insights, gene expression profiles were obtained from differentiating mESCs after 6 h and 24 h treatments with WS^®^1442 and MeOH eluate (**Figure 5A**). Genes regulated by both treatments were considered for gene ontology (signaling network) analyses which revealed a greater overlap after 24 h (30%) as opposed to 6 h (18%). Particularly, genes associated with cardiovascular development and function as well as cellular development, growth and proliferation were regulated (**Figure 5C**). This is expected, in line with the observed phenotypes (**Figure 1**) and characterizations of mesodermal and cardiovascular cell differentiation fates (**Figure 2**). The fact that mainly enzymes, kinases, and transcription factors were altered (**Figure 5C**) indicated strong effects on signaling transduction pathways.

From this transcriptome data, IPA-algorithms predicted the mechanistic involvement of distinct upstream effectors like tumor suppressor p53 which was activated after 6 h (z-score +2.20, **Figure 5D**). It is a key player in many biological processes such as apoptosis, genomic stress and stability, cellular aging, as well as stem and progenitor cell maintenance (Riley et al., 2008; Levine and Oren, 2009; Lin and Lin, 2017). Thus, upregulation of the p53 network could explain an immediate cellular response to WS^®^1442/MeOH eluate-caused stress besides promoting differentiation as opposed to maintaining a given developmental state (“stable expansion”). Stress- and inflammation-associated genes were also affected after 24 h (**Figure 5E**) explaining the upregulation of a “lipopolysaccharide (LPS)” network of genes (z-score +2.01). Therefore, it is conceivable that a significant part of the molecular mechanisms in WS^®^1442’s and MeOH eluate’s stimulation of cardiac differentiation is linked to an increased release of pro-inflammatory cytokines, eicosanoids, nitric oxide (NO), and/or reactive oxygen species (ROS) which exert a pro-differentiation effect. In general, such mechanisms have been demonstrated for ROS and NO in the context of mESC cardiac differentiation (Sauer et al., 2000; Bartsch et al., 2011). NO- and ROS-associated molecular mechanisms have already been proposed for *Crataegus* extracts in different cellular context (Koch and Chatterjee, 2000; Anselm et al., 2009). Stress-associated mechanisms were in agreement with data from cell viability assays which revealed decreased metabolic activity after 48 h treatment with WS^®^1442 and MeOH eluate compared to DMSO control (**Figure 4E**).

After 6 h of treatment, brain-derived neurotrophic factor (BDNF) was also predicted to be activated (z-score +1.95) as an upstream regulator. This is interesting because BDNF has been shown to exhibit several functions beyond the central nervous system. For example, BDNF overexpression is associated with an increased capillary density in heart tissue and reported to exert protective effects in coronary heart disease (Lorgis et al., 2010; Jiang et al., 2011; Kaess et al., 2015; Pius-Sadowska and Machaliński, 2017). Samal et al. (2015) showed that BDNF is increased in Sca-1^+^ progenitors from the failing heart and contributed to the cardiogenic potential of these cells *via* stimulation of cell migration (Samal et al., 2015). Additional potential immediate effectors of WS^®^1442 and MeOH eluate could be linked to the TGFβ superfamily of ligands, including nodal (e.g., *Lefty1* as a negative regulator of nodal) and bone morphogenetic protein (BMP) (e.g., *Lzts1*) which were significantly downregulated. These belong to the many well-known cardiopoietic signaling pathways that have been characterized for their spatio-temporal roles in heart morphogenesis, cardiomyocyte formation and specification (Noseda et al., 2011; Burridge et al., 2014; Paige et al., 2015; Spiering et al., 2015). Consistent with this data, the TGFβ-1 network was also found to be negatively regulated (z-score -2.28) after 24 h of treatment (**Figure 5E**). The requirement of TGFβ inhibition for effective cardiac differentiation has been shown by several groups, including our own work on a novel class of small molecular TGFβ inhibitors (Willems et al., 2012; Schade et al., 2012; Langle et al., 2019). Another downregulated signaling network after 24 h was FGF-2 (z-score -2.15) which is also well-recognized as a cardiogenic factor in stem cell differentiation and cardiac reprogramming from fibroblasts (Itoh et al., 2016). An interesting observation was the strong upregulation of *Aldh1a2* (5–7-fold, p < 0.0001) by both WS^®^1442 and MeOH eluate. *Aldh1a2* encodes for the retinaldehyde dehydrogenase 2 (Raldh2) which catalyzes the last step in the biosynthesis of retinoic acid (RA), i.e. the oxidation of retinaldehyde to RA. The pivotal role of RA in heart development and cardiomyogenesis is well-known (Moss et al., 1998; Ross et al., 2000). Many groups have reported that RA signaling contributes to cardiomyocyte specification with RA stimulation promoting an atrial over ventricular phenotype in differentiating murine and human ESCs (Gassanov et al., 2008; Zhang et al., 2011; Quaranta et al., 2018). A potential regulation of RA signaling by *Crataegus* extract is intriguing as the formation of atrial cardiomyocytes appeared to be favored over ventricular cells (see **Figure 4C**). However, the timeline of differentiation is not long enough in this setup to determine whether true subtype-specification is induced. Long-term culturing of differentiated ESC-derived cardiomyocytes and electrophysiological characterizations would be required.

Based on the transcriptome data, two plausible mechanisms for the differentiation-promoting activity of WS^®^1442 seemed to emerge: Early, stress-associated cellular events along with the perturbation of discrete developmental pathways, including the downregulation of TGFβ/BMP and FGF and the activation of BDNF- and RA-linked signaling. Future studies will have to dissect the activities of *Crataegus* extract, fractions/eluates, and ideally, further isolated compound classes on these pathways in cardiovascular progenitor cell populations.

As cardiovascular progenitors seem to be targeted, we aimed at investigating effects of WS^®^1442 and its bioactive fractions on physiologically relevant progenitor cell populations that have been characterized to reside within the heart (van Vliet et al., 2012; Broughton et al., 2018). The general role and impact of cardiac progenitor cells (CPCs) to cardiac regeneration after ischemic injury is controversial and has been challenged in recent years (Chien et al., 2019), at least regarding their contribution to *de novo* cardiomyocyte generation. Here, we used a cell population that has been proposed in the past decades as potential CPCs (i.e., Sca-1^+^ cells). However, it turned out to resemble rather endothelial cell phenotypes with minimal cardiomyogenic potential in tissue homeostasis and after myocardial infarction (Neidig et al., 2018; Zhang et al., 2018). Moreover, genetic lineage tracing of these Sca-1^+^ cells rather suggested a contribution to vasculogenesis in the murine heart (Vagnozzi et al., 2018).

This minimal capacity for cardio- and angiogenesis prompted us to assess function, differentiation, and proliferation of Sca-1^+^ progenitor cells (Sca-1-PCs) (Samal et al., 2012) from murine hearts *ex vivo* upon treatment with WS^®^1442, ultratetentate, or MeOH (**Figure 6A**). WS^®^1442 and MeOH eluate improved Sca-1-PC attachment but had no effects on proliferation rates (**Figure 6B**). Ultraretentate treatment exhibited a more modest effect. In contrast to WS^®^1442, Sca-PC proliferation rates were slowed down after 24–48 h with ultraretentate and MeOH eluate. Flow cytometry analysis of WS^®^1442- and MeOH eluate-treated Sca-PCs after 72-h treatments revealed no significant change in the percentage of Sca-1^+^ cells supporting a stabilizing, pro-cardiovascular cell-fate activity (**Figure 6C**). WS^®^1442 induced capillary-like tubule formation of Sca-PCs in a concentration-dependent fashion (**Figure 6D**, left) while MeOH eluate-treatment inhibited this phenotype (**Figure 6D**, right). This is interesting because our transcriptome data on differentiating mESCs suggested BDNF as a putative upstream regulator which has been described to induce mobilization, angiogenesis, and cardiogenic potential of Sca-1^+^ progenitor cells (Samal et al., 2015). These opposing effects of WS^®^1442 and MeOH eluate further underline that isolated, primary Sca-1^+^ cardiovascular progenitors represent a distinct cell type compared to cardiac progenitors from mESC differentiation. Future studies will have to shed light on cardiovascular cell-fates of such Sca-PCs after long-term exposure to WS^®^1442 and MeOH eluate and functional consequences thereof. This is particularly appealing in view of numerous studies that suggest proangiogenic therapy might be promising for patients after acute myocardial infarction (Cochain et al., 2013).

**Figure 7:**
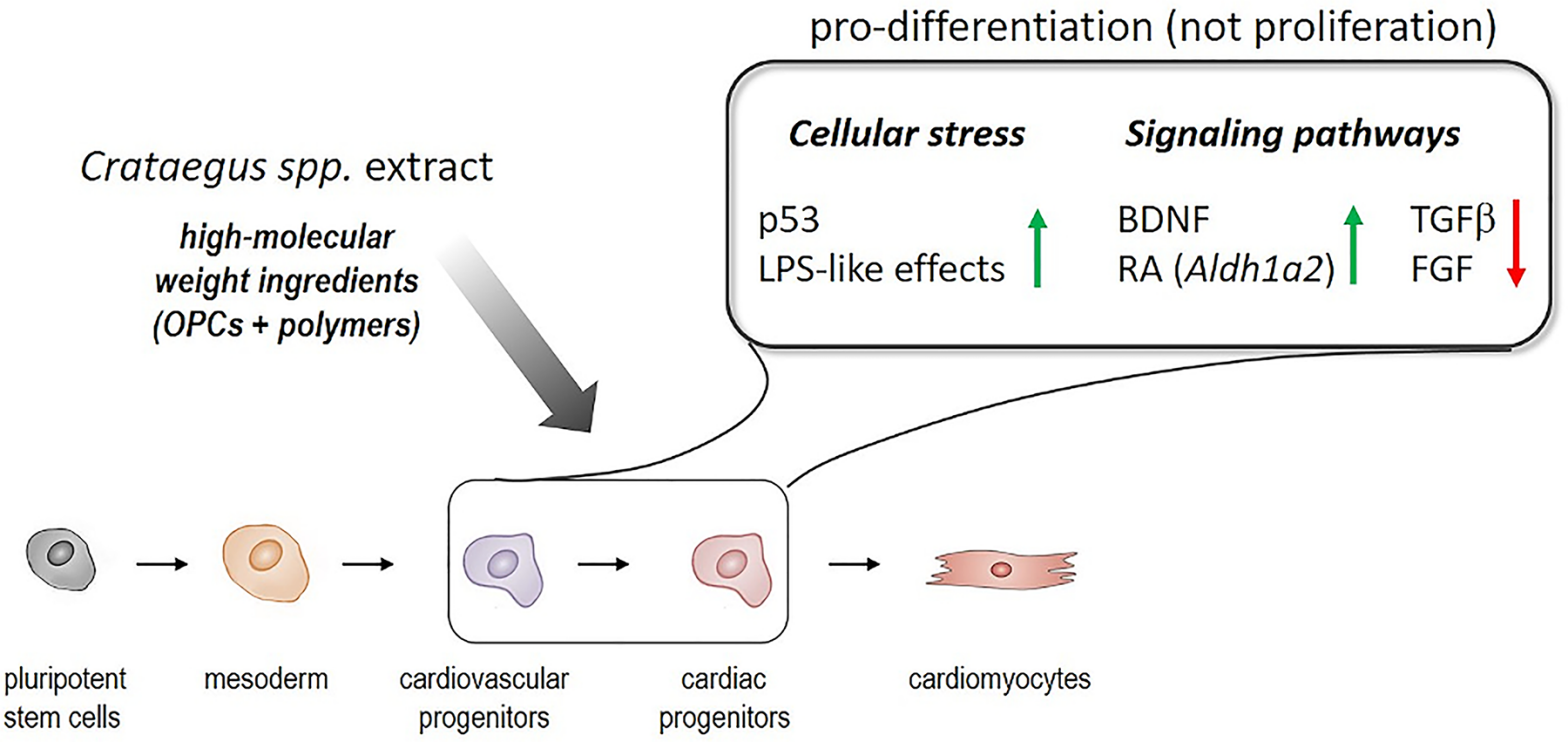
Summary of the differentiation-promoting profile of *Crataegus* extract WS^®^1442 on cardiovascular progenitor cells.

In conclusion, the underlying mechanisms for a pro-differentiation profile of WS^®^1442 on cardiovascular progenitor cells involve rather unspecific stress-associated effects along with the perturbation of distinct signaling pathways, such as TGFβ, FGF, retinoic acid, and BDNF (**Figure 7**). It will be critical to address to which extent WS^®^1442 and its bioactive fractions/eluates contribute to cardiogenic and/or angiogenic potential *in vivo* under physiological conditions as well as after ischemic injury, implicating an unprecedented pharmacology of this traditional medicinal plant. In this regard, it will be interesting to test whether the putative high-molecular weight ingredients (e.g., OPC clusters DP3–6 and carbohydrate polymers) show efficacy after oral administration. Independent from such bioavailability studies, further bioassay-guided fractionations—employing both mESC-derived cardiovascular progenitors and primary Sca-PCs—will be highly attractive to discriminate cardiogenic from angiogenic *Crataegus* components and elucidate their mode-of-action on a molecular level. Since there is a growing agreement in the field that pre-existing cardiomyocytes likely represent the major contributors to *de novo* cardiomyocyte formation (Foglia and Poss, 2016), it will be important to further explore *Crataegus* extracts in the context of cardiomyocyte formation.

## Data Availability Statement

The transcriptome datasets generated for this study can be found in the NCBI GEO database (accession number GSE133572).

## Author Contributions

DS is responsible for conceptualization, project management, data interpretation, discussions, and manuscript writing. JH, KW, SK, EK, JS, AH, and CG co-wrote the manuscript. JH generated, processed, and analyzed data shown in **Figures 1**, **2**, **4**, **5**, and **S5**. CG supported all flow cytometry experiments. CE and EK provided WS^®^1442, performed fractionations, and provided the respective analytical data depicted in **Figure 2A, B**. JS, NS, and AH produced data shown in **Figures 3** and **S1**–**S4**, **Tables S1**–**S3**. KW and SK generated, processed, and analyzed data shown in **Figure 6**. EW and MM provided data shown in **Figure 1A**.

## Funding

For financial support we acknowledge Dr. Willmar Schwabe GmbH & Co. KG (Karlsruhe, Germany) who funded parts of the herein presented work. We also thank the Federal Ministry of Science and Education (BMBF) for funding (grant 1316053).

## Conflict of Interest

The authors declare that this study received funding from Dr. Willmar Schwabe GmbH & Co. KG to conduct parts of the herein presented work (i.e., data shown in **Figures 1**, **2**, **3**, **4** and **5**). CE and EK performed fractionations of WSR1442 and provided the respective analytical data depicted in **Figures 2A, B**. The funder had no role in study design, data collection and analysis, decision to publish, or preparation of the manuscript, except for proof-reading (EK and CE) of the final version.

## Abbreviations

ANF, atrial natriuretic factors; BDNF, brain-derived neurotrophic factor; BMP, bone morphogenetic protein; cTnT, cardiac Troponin T; DP, degree of polymerization; ESCs, embryonic stem cells (murine, mESCs; human, hESCs); EtOH eluate, ethanol eluate; FGF, fibroblast growth factor; MeOH eluate, methanol eluate; MHC, myosin heavy chain; OPCs, oligomeric procyanidines; PDGFβ, platelet-derived growth factor subunit β; RA, retinoic acid; Raldh2, retinaldehyde dehydrogenase 2; ROS, reactive oxygen species; Sca-1, stem cells antigen-1; Sca-PC, Sca-1+ progenitor cells; TGFβ, transforming growth factor β; WT, wild-type.

## Supplementary Material

Supplementary Material contains an overview on chemical structures of *Crataegus* ingredients and tested compounds (**Figure S1**), detailed LC-FD- and LC-MS-based phytochemical analyses of WS^®^1442 and its fractions/eluates (**Figures S2**–**S5**, **Tables S1**–**S3**), Log2(fold change)-plots of all commonly regulated genes upon WS^®^1442 and MeOH eluate treatments during mESC differentiation (**Figure S6**), and representative videos of beating mESC-derived cardiomyocytes upon treatment with WS^®^1442 (**Video S1**) and MeOH eluate (**Video S2**)
