# Supplementary material for: Crataegus Extract WS®1442 Stimulates Cardiomyogenesis and Angiogenesis From Stem Cells: A Possible New Pharmacology for Hawthorn?
Source: Front Pharmacol. 2019 Nov 27;10:1357. doi: 10.3389/fphar.2019.01357 (PMC6902660; doi:10.3389/fphar.2019.01357)
Supplement: Supplementary file 1 [file DataSheet_1.pdf]

Supplementary Material

Crataegus Extract WS®1442 Stimulates Cardiomyogenesis and Angiogenesis From Stem Cells: A Possible New Pharmacology for Hawthorn?

Jonas Halver[1], Kristin Wenzel[2,3], Jandirk Sendker[4], Carmen Carrillo-Garcia[5], Clemens A. J. Erdelmeier[6], Erik Willems[7], Mark Mercola[7], Nico Symma[4], Stephanie Köneman[2,3], Egon Koch,[6] Andreas Hensel[7], Dennis Schade[1,5,8*]

Content

1) **Chemical structures of *Crataegus spp.* ingredients and tested substances** **p2**

2) **LC-FD- and LC-MS-based phytochemical analyses** **p3-12**

- Figure S2: HPLC-FD chromatogram of WS®1442
- Figure S3: Extracted ion chromatograms for OPC DP2-12 of WS®1442
- Figure S4: Correlation between OPC DP2-8 signals of WS®1442 as observed by HPLC-FD and LC-MS
- Table S1: LC-MS data on OPC and related compounds, assorted by DP
- Table S3: LC-MS data on OPC (DP1 only) and other compounds
- Table S3: Identification data to Table S2
- Figure S5: ESI mass spectrum of OPC hexoside DP2

3) **Transcriptome analyses** **p12**

- Figure S6: Log2(FC)-plot of all commonly regulated genes upon WS®1442 and MeOH eluate treatments during mESC differentiation

4) **Supplementary video** **p13**

- Video S1: Beating cardiomyocytes on day 11 after WS®1442 treatment (d4-6)
- Video S2: Beating cardiomyocytes on day 11 after MeOH eluate treatment (d4-6)

5) **References** **p14**

1) Chemical structures of *Crataegus spp.* ingredients and tested substances

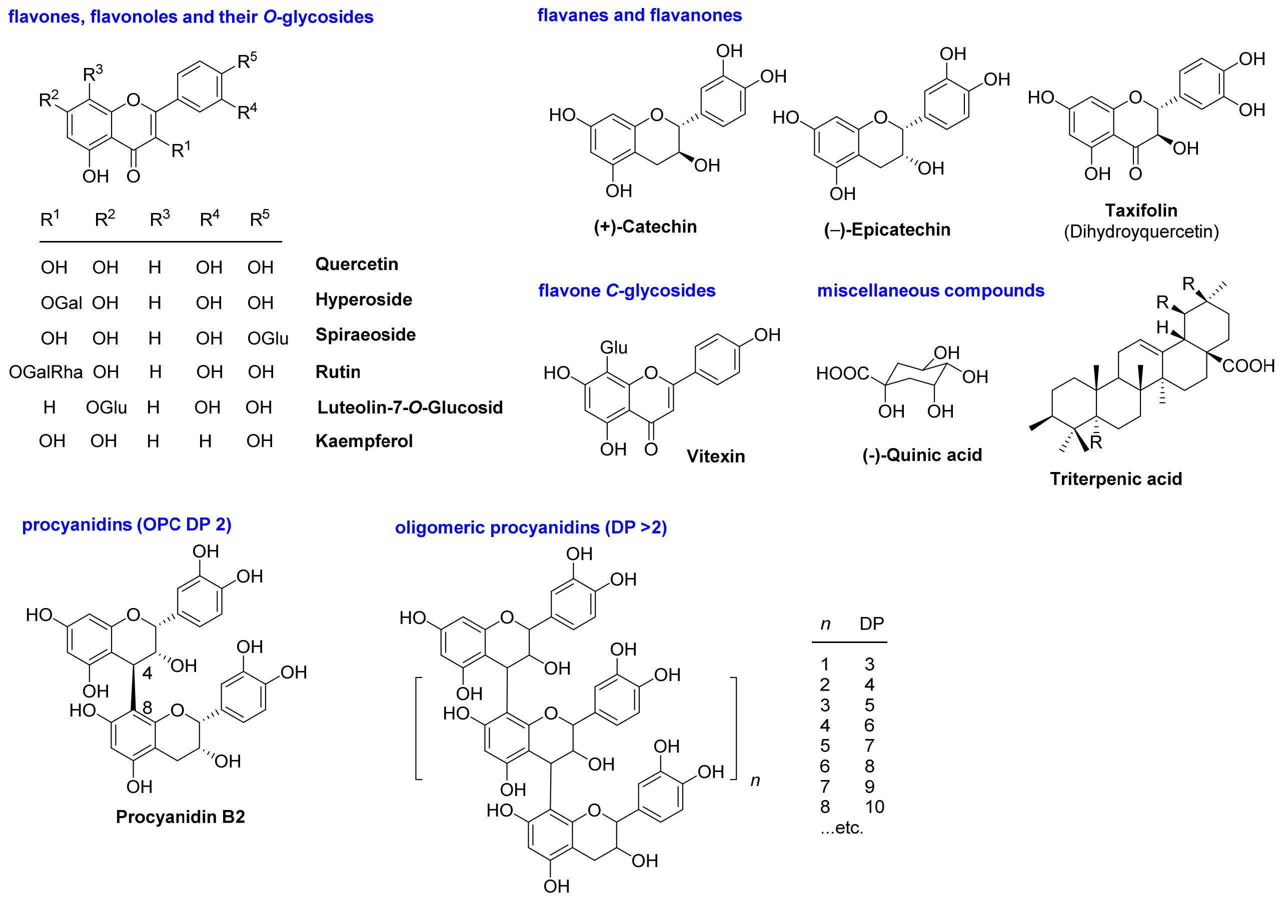

Figure S1. Chemical structures of *Crataegus spp.* ingredients and tested substances.

2) LC-FD- and LC-MS-based phytochemical analyses

WS®1442 and its fractions were characterized by LC-MS using RP18 chromatography and by HPLC-FD using a diole stationary phase in HILIC mode. The latter method, adapted from previous work,[1] separates OPC according to their degree of polymerization (DP). OPC up to DP12 were detected in WS1442 along with a polymer peak, which may be assumed to contain OPC constituted of more than twelve flavan-3-ol units (Figure S2).

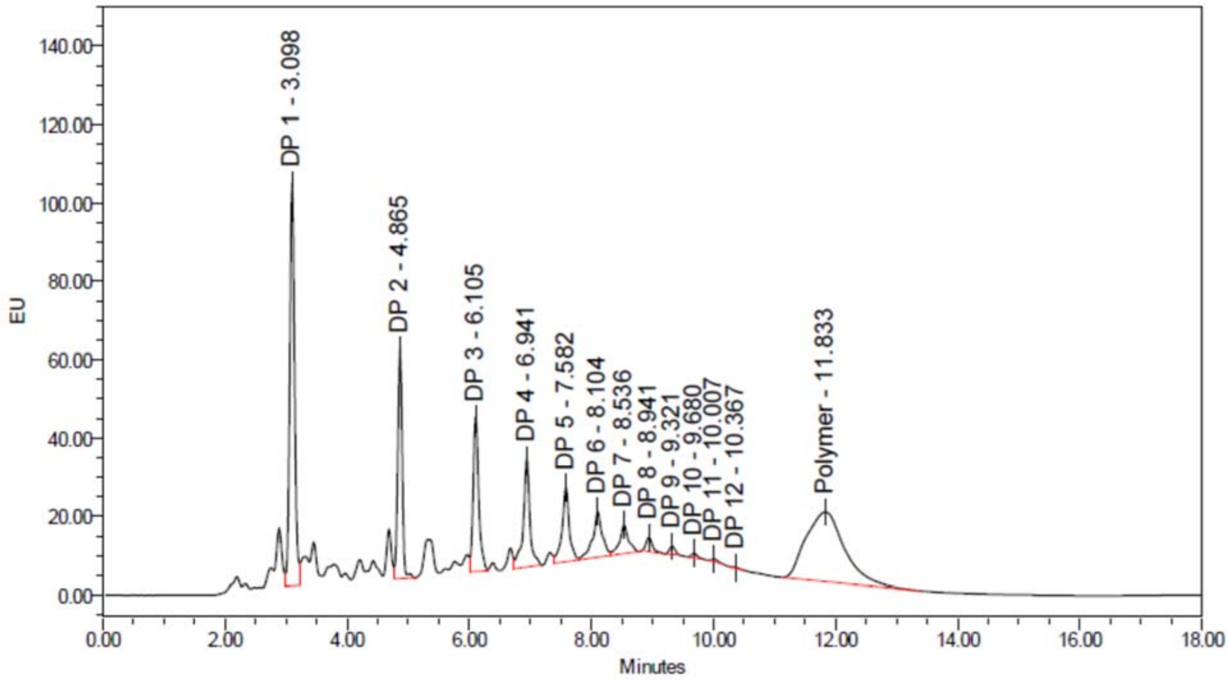

Figure S2. Representative HPLC-FD chromatogram of WS®1442.

LCMS was used to analyze the samples for OPC, OPC-related constituents (Table S1), and other constituents. OPC isotope patterns were observable by LCMS up to DP8, whereas only diffuse increases in the extracted ion chromatograms (EIC) adumbrated the presence of DP9–12 (Figure S3). A non-linear relationship between the OPC patterns observed by LCMS and HPLC-FD indicated an exponential loss of LCMS-sensitivity with increasing DP (Figure S4). However, this bias does not significantly affect the OPCs' ranks (r_S = 0.93) and thus the fundamental interpretability of the OPC patterns.

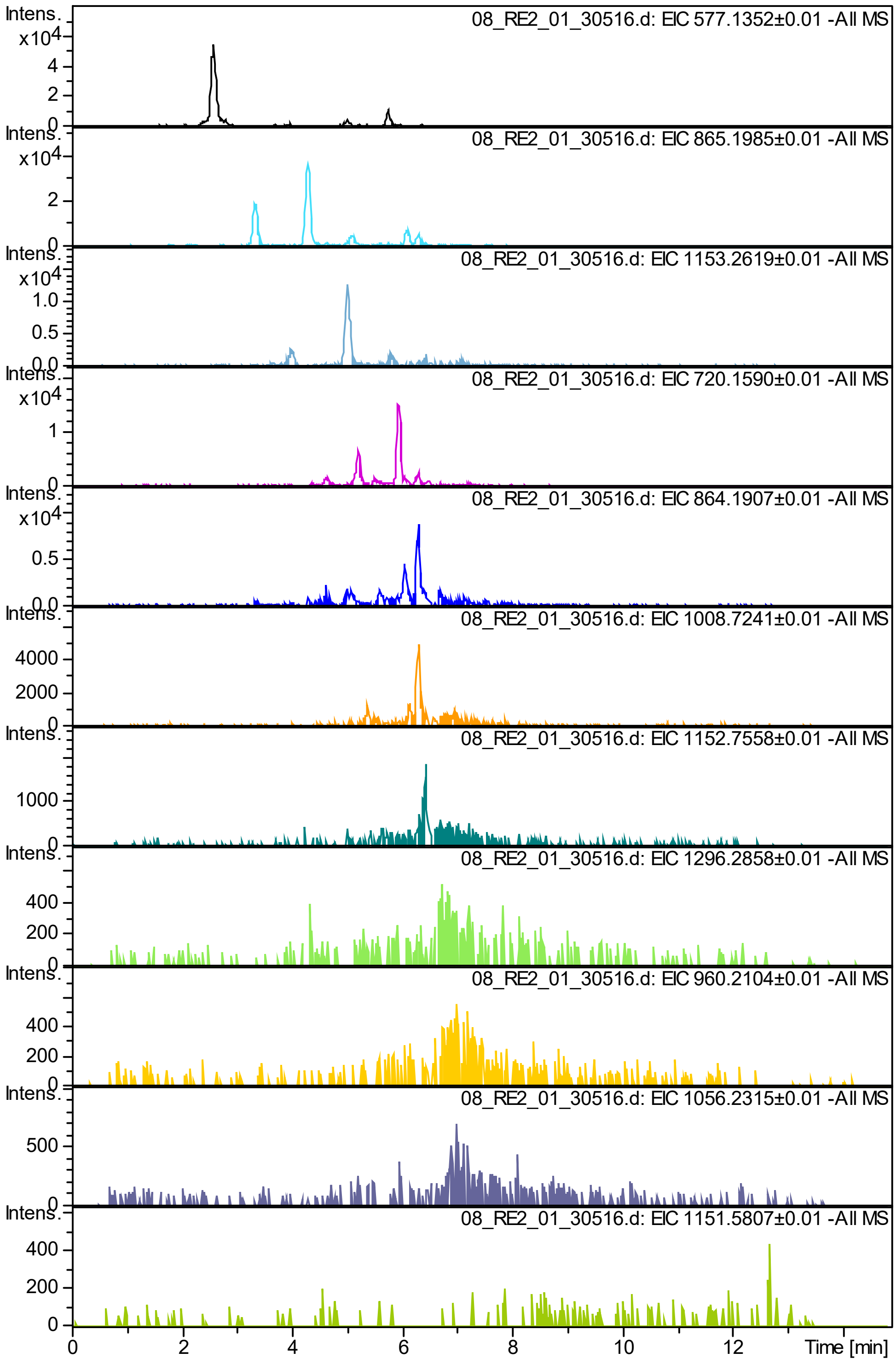

Figure S3. Extracted ion chromatograms (EIC) for OPC DP2-12 of WS®1442.

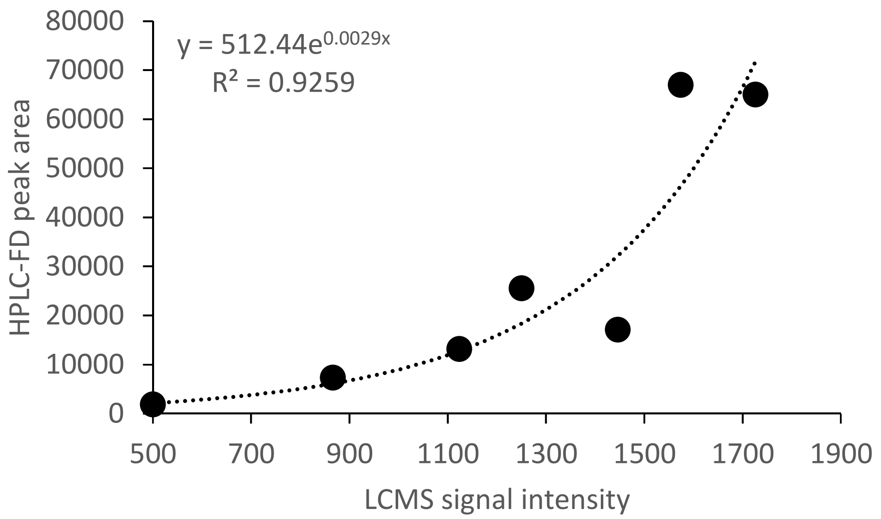

Figure S4. Correlation between OPC DP2-8 signals of WS®1442 as observed by HPLC-FD and LC-MS.

Table S1. LC-MS data on OPC and related compounds, assorted by degree of polymerization (DP)

DP	class	ion formula	t_R / min	*m/z*	error / mDa	mΣ	intensity of the most intense isotope signal					
							WS1442	water	ethanol	methanol	acetone	ultra-retentate
1	OPC	$[C_{15}H_{13}O_6]^-$	4.823	289.0715	–0.3	2.4	33653		137434	7300	6531	2893
1	OPC	$[C_{15}H_{13}O_6]^-$	4.521	289.0719	–0.2	5.5	2235		15738	624	694	
1	hexoside	$[C_{21}H_{23}O_{11}]^-$	3.879	451.1229	1.7	27.6	1288	1202	1800			
1	hexoside	$[C_{21}H_{23}O_{11}]^-$	4.178	451.1236	1.0	132.9	932	514	1112			
1	cinchonain	$[C_{24}H_{19}O_9]^-$	6.240	451.1021	1.4	1.0	9422		45333	469	268	
1	cinchonain	$[C_{24}H_{19}O_9]^-$	5.720	451.1022	1.3	7.9	5949		31197	296	260	
2	OPC	$[C_{30}H_{25}O_{12}]^-$	2.568	577.1316	2.2	14.9	53623		147547	78468	23953	18961
2	OPC	$[C_{30}H_{25}O_{12}]^-$	5.746	577.1344	0.8	10.6	11374		1132	22427	1413	943
2	hexoside (see Fig. S4)	$[C_{36}H_{35}O_{17}]^-$	1.481	739.1845	0.6	12.8	7722		64703	2633	1060	4951
2	cinchonain	$[C_{39}H_{31}O_{15}]^-$	5.423	739.1629	–2.5	5.3	8008		4690	38713	535	1232
2	cinchonain	$[C_{39}H_{31}O_{15}]^-$	4.895	739.1641	1.7	1.6	12096		9278	46550	520	1827
3	OPC	$[C_{45}H_{37}O_{18}]^-$	5.086	865.1954	–3.1	22.2	5020			10908	1285	1336
3	OPC	$[C_{45}H_{37}O_{18}]^-$	4.289	865.1965	–2.0	12.1	35762		3485	115830	12756	22389
3	OPC	$[C_{45}H_{37}O_{18}]^-$	6.081	865.1970	–1.6	7.6	7268			11757	950	1912
3	OPC	$[C_{45}H_{37}O_{18}]^-$	3.345	865.2007	2.2	13.1	18935			36629	1839	4501
3	hexoside	$[C_{51}H_{47}O_{23}]^-$	2.655	1027.2417	–1.8	17.0	1137			5905		1621
3	hexoside	$[C_{51}H_{47}O_{23}]^-$	3.320	1027.2426	–3.7	57.7	613			1701		669
3	hexoside	$[C_{51}H_{47}O_{23}]^-$	2.213	1027.2463	–3.0	27.8	1878		1039	7195		1909
3	cinchonain	$[C_{54}H_{43}O_{21}]^-$	6.595	1027.2247	3.5	9.2	3124		2099	8244		
3	cinchonain	$[C_{54}H_{43}O_{21}]^-$	6.228	1027.2251	–3.5	3.8	2787		374	8217		2359
4	OPC	$[C_{60}H_{49}O_{24}]^-$	5.792	1153.2556	–6.3	115.6	2054			3436	1463	
4	OPC	$[C_{60}H_{49}O_{24}]^-$	4.990	1153.2608	–1.1	14.4	12386			21371		6035
4	OPC	$[C_{60}H_{49}O_{24}]^-$	3.978	1153.2640	–2.0	29.8	2629			4919		1104
4	hexoside	$[C_{66}H_{58}O_{29}]^{2-}$	2.789	657.1499	1.5	152.2	479			1062		1005
4	hexoside	$[C_{66}H_{58}O_{29}]^{2-}$	3.306	657.1526	1.1	31.9	662			1705		776
4	cinchonain	$[C_{69}H_{55}O_{27}]^-$	6.540	1315.3004	–9.0	98.2	1414		4929	1359		
5	OPC	$[C_{75}H_{60}O_{30}]^{2-}$	6.309	720.1548	–4.2	82.0	2441			3149	416	1427
5	OPC	$[C_{75}H_{60}O_{30}]^{2-}$	4.619	720.1552	3.8	84.7	1704			2602	300	1375
5	OPC	$[C_{75}H_{60}O_{30}]^{2-}$	5.928	720.1584	–0.7	36.7	14981			17014	1322	8004
5	OPC	$[C_{75}H_{60}O_{30}]^{2-}$	5.194	720.1599	–0.9	65.3	6363			7973	690	2976
5	hexoside	$[C_{81}H_{70}O_{35}]^{2-}$	4.574	801.1810	3.0	82.3	571			708		707
5	hexoside	$[C_{81}H_{70}O_{35}]^{2-}$	3.820	801.1818	–0.4	223.2				798		626
5	cinchonain	$[C_{84}H_{66}O_{33}]^{2-}$	6.790	801.1688	0.8	80.2	1078			3247		1398
5	cinchonain	$[C_{84}H_{66}O_{33}]^{2-}$	7.253	801.1702	6.1	96.4	1225			3296		1157
6	OPC	$[C_{90}H_{72}O_{36}]^{2-}$	6.048	864.1835	–7.2	193.9	4516			3791		1986
6	OPC	$[C_{90}H_{72}O_{36}]^{2-}$	6.280	864.1886	–2:1	52.2	8624			6508	912	3433
6	cinchonain	$[C_{99}H_{78}O_{39}]^{2-}$	7.414	945.1993	7.1	82.3	600			1359		713
6	cinchonain	$[C_{99}H_{78}O_{39}]^{2-}$	6.959	945.2036	–7.2	176.6	341					735
7	OPC	$[C_{105}H_{84}O_{42}]^{2-}$	6.127	1008.2152	7.2	115.4	1186			1426		
7	OPC	$[C_{105}H_{84}O_{42}]^{2-}$	6.287	1008.2172	–5.2	56.9	4859			3761	428	1924
7	OPC	$[C_{105}H_{84}O_{42}]^{2-}$	5.360	1008.2229	–0.5	214.7	1249					
8	OPC	$[C_{120}H_{96}O_{48}]^{2-}$	6.430	1152.2566	–2.5	138.3	1819			1355		

Data for DP1 compounds were in part not accessible by method A (mass range, *t*R) and thus completely taken from data of method B.

Table S2. LC-MS data on OPC (DP1 only) and other compounds.

tentative assignment	ion formula	t_R/min	*m/z*	Δ *m/z*	mΣ	intensity of the most intense isotope signal					
						WS1442	water	ethanol	methanol	acetone	ultra-retentate
quinic acid	$[C_7H_{11}O_6]^-$	1.34	191.0209	1.2	8.2	17254	18193				
kaempferol	$[C_{15}H_9O_6]^-$	8.54	285.0389	−1.5	16.5	590		2372			
caffeoyl threonic acid	$[C_{13}H_{13}O_8]^-$	4.94	297.0624	0.8	13.9	5245	6417	368			
quercetin	$[C_{15}H_9O_7]^-$	7.74	301.0345	−0.5	3.4	5625		34924	1380	1550	
O-methylated quercetin or isomeric flavonoid	$[C_{16}H_{11}O_7]^-$	8.03	315.0499	−1.1	14.1	1502		9619			
monocumaroyl quinic acid	$[C_{16}H_{17}O_8]^-$	5.12	337.0935	−0.6	16.4	10290	8645	1025			
monocumaroyl quinic acid	$[C_{16}H_{17}O_8]^-$	4.48	337.0938	0.9	13.1	7136	6973	547			
monocaffeoyl quinic acid	$[C_{16}H_{17}O_9]^-$	3.82	353.0885	−0.3	2.5	19167	19081	1354			
monocaffeoyl quinic acid	$[C_{16}H_{17}O_9]^-$	4.71	353.0886	0.2	2.5	54476	55018	4848	2136		950
monocaffeoyl quinic acid	$[C_{16}H_{17}O_9]^-$	4.62	353.0895	1.2	6.3	13674	15078	1872		1052	489
apigenin-*C*-hexoside (vitexin)	$[C_{21}H_{19}O_{10}]^-$	5.44	431.0988	0.4	2.7	9173		49500	391	232	688
naringenin hexoside	$[C_{21}H_{21}O_{10}]^-$	5.72	433.1136	−4.0	13.0	2937		16955			
kaempferol *O*-hexoside (kaempferol 3-galactoside)	$[C_{21}H_{19}O_{11}]^-$	6.10	447.0925	−0.8	3.2	3979	2035	21924			
eriodictyol *O*-hexoside	$[C_{21}H_{21}O_{11}]^-$	5.36	449.1085	−0.5	8.5	2972		14635			
quercetin *O*-hexoside (hyperoside, spiraeoside)	$[C_{21}H_{19}O_{12}]^-$	5.83	463.0885	−0.8	3.4	48195	14275	66935	2930	1547	3719
steroid	$[C_{23}H_{45}O_9]^-$	10.43	465.3068	0.1	26.4	1279		4603			
triterpene	$[C_{30}H_{47}O_5]^-$	9.13	487.3393	3.3	12.0	724		3359			
triterpene	$[C_{30}H_{47}O_5]^-$	9.61	487.3422	−0.7	7.3	7384	377	16839	458		
cumaroyl caffeoyl quinic acid	$[C_{25}H_{23}O_{11}]^-$	6.97	499.1233	1.3	23.9	753		5392			
dicaffeoyl quinic acid	$[C_{25}H_{23}O_{12}]^-$	6.52	515.1177	1.8	3.9	6924		50551	1669		443
dicaffeoyl quinic acid	$[C_{25}H_{23}O_{12}]^-$	6.32	515.1178	1.7	3.4	1576		15750	367		366
dicaffeoyl quinic acid	$[C_{25}H_{23}O_{12}]^-$	6.24	515.1181	−1.4	8.0	3415		17294	376		307
triterpene acid	$[C_{30}H_{45}O_7]^-$	9.04	517.3151	−2.0	6.5	1266		9501			
steroid pentoside	$[C_{28}H_{47}O_{11}]^-$	9.13	559.3101	2.3	15.1	3291		5812			
vitexin-*O*-pentoside (vitexin-2"-*O*-*β*-xylopyranoside)	$[C_{26}H_{27}O_{14}]^-$	5.13	563.1412	−0.2	7.9	25312	23467	11756			3410
iso(schaftoside)	$[C_{26}H_{27}O_{14}]^-$	5.00	563.1415	0.2	5.6	6189	4469	3262			649
vitexin-*O*-pentoside (vitexin-2"-*O*-*β*-xylopyranoside)	$[C_{26}H_{27}O_{14}]^-$	5.28	563.1419	−0.8	5.8	37711	30012	80614	1172		14458
vitexin-*O*-rhamnopyranoside	$[C_{27}H_{29}O_{14}]^-$	5.23	577.1573	0.1	0.9	75025	73137	59440	2045	662	32765
tricoumaroyl spermidine	$[C_{34}H_{36}N_3O_6]^-$	7.34	582.2606	−0.4	6.8	11133		29088	481		
rutin	$[C_{27}H_{29}O_{16}]^-$	5.65	609.1469	1.1	4.9	20697		75903	1165	465	2809
acetylated vitexin-*O*-desoxyhexoside	$[C_{29}H_{31}O_{15}]^-$	5.50	619.1666	0.3	9.6	7161	3803	32781			2444
acetylated vitexin-*O*-desoxyhexoside	$[C_{29}H_{31}O_{15}]^-$	5.80	619.1688	−0.6	4.0	46645	42805	60318	727	288	16825
steroid (C_{22}) dipentoside	$[C_{32}H_{59}O_{16}]^-$	9.26	699.3805	0.4	10.2	2152		13813			473

Table S3. Identification data to Table S2.

tentative assignment	ion formula	t_R/min	*m/z*	Δ *m/z*	mΣ	MS2, rel. I (40 eV)	reference
quinic acid	$[C_7H_{11}O_6]^-$	1.34	191.0209	1.2	8.2	–	
kaempferol	$[C_{15}H_9O_6]^-$	8.54	285.0389	–1.5	16.5	–	
caffeoyl threonic acid	$[C_{13}H_{13}O_8]^-$	4.94	297.0624	0.8	13.9	135.0370, 100<br>105.0322, 37	
quercetin	$[C_{15}H_9O_7]^-$	7.74	301.0345	–0.5	3.4	–	
O-methylated quercetin or isomeric flavonoid	$[C_{16}H_{11}O_7]^-$	8.03	315.0499	–1.1	14.1	300.0337, 34<br>271.0307, 20<br>255.0184, 36<br>243.0278, 32<br>229.0097, 21<br>227.0317, 28<br>216.0410, 24<br>183.0457, 15<br>174.0231, 37<br>165.9911, 53<br>157.0256, 45<br>139.0017, 36<br>109.9997, 100	
monocumaroyl quinic acid	$[C_{16}H_{17}O_8]^-$	5.12	337.0935	–0.6	16.4	191.0735, 11<br>163.0361, 49<br>155.0170, 7<br>119.0492, 100	
monocumaroyl quinic acid	$[C_{16}H_{17}O_8]^-$	4.48	337.0938	0.9	13.1	191.0657, 18<br>163.0405, 72<br>155.0326, 15<br>119.0501, 100	
monocaffeoyl quinic acid	$[C_{16}H_{17}O_9]^-$	3.82	353.0885	–0.3	2.5	191.0735, 84<br>179.0358, 24<br>173.0410, 5<br>161.0254, 8<br>135.0439, 100<br>105.0313, 4	mzcloud
monocaffeoyl quinic acid	$[C_{16}H_{17}O_9]^-$	4.71	353.0886	0.2	2.5	191.0560. 100<br>179.0371, 2<br>173.0445, 5<br>161.0236, 3<br>135.0454. 8	mzcloud
monocaffeoyl quinic acid	$[C_{16}H_{17}O_9]^-$	4.62	353.0895	1.2	6.3	191.0549, 64<br>179.0364, 24<br>173.0429, 48<br>161.0182, 7<br>155.0398, 8<br>135.0465, 100<br>93.0377, 26<br>85.0309, 13	mzcloud
apigenin *C*-hexoside (vitexin)	$[C_{21}H_{19}O_{10}]^-$	5.44	431.0988	0.4	2.7	341.0725, 6<br>323.0604, 9<br>311.0572, 84<br>293.0478, 6<br>283.0593, 100<br>268.0371, 5<br>191.0288, 5<br>149.0237, 5	
naringenin hexoside	$[C_{21}H_{21}O_{10}]^-$	5.72	433.1136	–4.0	13.0	341.0705, 16<br>311.0521, 16<br>283.0619, 24<br>271.0624, 93<br>227.0721, 7<br>177.0133, 17<br>167.0322, 13<br>151.0021, 100<br>119.0480, 40<br>107.0139, 9<br>93.0266, 7	mzcloud

kaempferol *O*-hexoside<br>(kaempferol 3-galactoside)	$[C_{21}H_{19}O_{11}]^-$	6.10	447.0925	–0.8	3.2	285.0385, 42<br>284.0329, 100<br>255.0297, 55<br>227.0353, 33<br>150.9991, 5	mzcloud
eriodictyol *O*-hexoside	$[C_{21}H_{21}O_{11}]^-$	5.36	449.1085	–0.5	8.5	287.0528, 12<br>175.0030, 5<br>151.0026, 100<br>135.0433, 41<br>125.0219, 3	mzcloud
quercetin *O*-hexoside<br>(hyperoside, spiraeoside)	$[C_{21}H_{19}O_{12}]^-$	5.83	463.0885	–0.8	3.4	301.0326, 31<br>300.0271, 100<br>283.0258, 2<br>271.0237, 18<br>255.0294, 9<br>243.0306, 2<br>277.0309, 1<br>211.0415, 1<br>190.9938, 1<br>178.9972, 4<br>162.9982, 1<br>151.0024, 5	mzcloud
steroide	$[C_{23}H_{45}O_{9}]^-$	10.43	465.3068	0.1	26.4	255.2326, 100<br>181.0708, 2<br>163.0600, 4	
triterpene	$[C_{30}H_{47}O_{5}]^-$	9.13	487.3393	3.3	12.0	487.3350, 100<br>277.2154, 50	
triterpene	$[C_{30}H_{47}O_{5}]^-$	9.61	487.3422	–0.7	7.3	487.3433, 100<br>469.3318, 47<br>443.3584, 4<br>427.3071, 5<br>423.3243, 5<br>407.3322, 4<br>277.2190, 55<br>163.0603, 3<br>101.0254, 3	
cumaroyl caffeoyl quinic acid	$[C_{25}H_{23}O_{11}]^-$	6.97	499.1233	1.3	23.9	353.0826, 2<br>191.0556, 20<br>179.0325, 29<br>173.0443, 100<br>163.0371. 16<br>161.0239, 6<br>155.0332, 6<br>137.0187, 7	
dicaffeoyl quinic acid	$[C_{25}H_{23}O_{12}]^-$	6.52	515.1177	1.8	3.9	353.0841, 6<br>191.0543, 37<br>179.0331, 81<br>173.0437, 100<br>161.0244, 4<br>155.0343, 7<br>135.0434, 15	
dicaffeoyl quinic acid†	$[C_{25}H_{23}O_{12}]^-$	6.32	515.1178	1.7	3.4	353.0884, 2<br>341.0684, 4<br>191.0544, 100<br>179.0340, 75<br>173.0446, 8<br>161.0226, 6<br>155.0355, 3<br>135.0443,19	
dicaffeoyl quinic acid†	$[C_{25}H_{23}O_{12}]^-$	6.24	515.1181	–1.4	8.0		
triterpene acid	$[C_{30}H_{45}O_{7}]^-$	9.04	517.3151	–2.0	6.5	517.3158, 100<br>499.2960, 4<br>473.3394, 13<br>455.3115, 29<br>439.2950, 7	
steroid pentoside	$[C_{28}H_{47}O_{11}]^-$	9.13	559.3101	2.3	15.1	277.2152, 100<br>253.0915, 14<br>161.0321, 4<br>101.0256, 5	
vitexin-*O*-pentoside<br>(vitexin-2''-*O*-*β*-xylopyranoside)	$[C_{26}H_{27}O_{14}]^-$	5.13	563.1412	–0.2	7.9	563.1391, 3<br>413.0926, 29	

						353.0678, 3<br>341.0695, 5<br>335.0569, 3<br>311.0527, 16<br>293.0476, 100	
iso(schaftoside)	$[C_{26}H_{27}O_{14}]^{-}$	5.00	563.1415	0.2	5.6	563.1434, 44<br>503.1318, 12<br>474.0974, 23<br>473.1070, 15<br>443.0946, 46<br>413.0928, 14<br>395.0892, 14<br>383.0777, 100<br>353.0706, 61<br>325.0721, 11	Hao et al., 2018
vitexin-*O*-pentoside<br>(vitexin-2''-*O*-*β*-xylopyranoside)	$[C_{26}H_{27}O_{14}]^{-}$	5.28	563.1419	–0.8	5.8	413.0862, 21<br>323.0548, 3<br>311.0546, 12<br>293.0468, 100	
vitexin- or saponaretin *O*-rhamnopyranoside	$[C_{27}H_{29}O_{14}]^{-}$	5.23	577.1573	0.1	0.9	413.0868, 33<br>311.0570, 14<br>293.0430, 100	
tricoumaroyl spermidine	$[C_{34}H_{36}N_{3}O_{6}]^{-}$	7.34	582.2606	–0.4	6.8	502.7001, 3<br>462.2035, 34<br>436.2163, 7<br>419.1837, 6<br>342.1452, 88<br>316.1646, 16<br>299.1404, 6<br>145.0288, 35<br>119.0497, 100	
rutin	$[C_{27}H_{29}O_{16}]^{-}$	5.65	609.1469	1.1	4.9	609.1443, 23<br>300.0274, 100<br>178.9969, 3<br>151.0041, 2	mzcloud
acetylated vitexin-*O*-desoxyhexoside	$[C_{29}H_{31}O_{15}]^{-}$	5.50	619.1666	0.3	9.6	619.1507, 1<br>577.1534, 1<br>559.1493, 1<br>499.1234, 5<br>457.1079, 2<br>455.0934, 1<br>431.0885, 1<br>413.0870, 43<br>395.0617, 1<br>377.0747, 1<br>365.0613, 1<br>353.0735, 2<br>341.0697, 2<br>335.0574, 2<br>323.0561, 6<br>311.0518, 11<br>293.0450, 100<br>282.0457, 4<br>269.0445, 5	
acetylated vitexin-*O*-desoxyhexoside	$[C_{29}H_{31}O_{15}]^{-}$	5.80	619.1688	–0.6	4.0	619.1441, 1<br>499.1251, 4<br>457.1387, 1<br>413.0872, 49<br>341.0680, 2<br>311.0552, 12<br>293.0451, 100<br>282.0486, 2<br>269.0445, 1	

steroid (C_{22}) dipentoside	$[C_{32}H_{59}O_{16}]^-$	9.26	699.3805	0.4	10.2	415.1372, 40<br>397.1348, 100<br>361.1019, 3<br>255.2319, 49<br>235.0820, 13<br>179.0561, 13<br>161.0472, 9<br>119.0347, 19<br>101.0174, 4	

† Insufficient chromatographic resolution of chromatographic peaks, MS2 data result from a mixture of isomeric precursor ions.

Cinchonains of DP1–6 were detectable in WS®1442 and its fractions/eluates, with estimated relative amounts of 5–10 % of their respective OPC for DP3–6 but 30–40 % for DP1–2. OPC hexosides have been tentatively described for *Crataegus spp.* on the basis of HRMS data (DP1–2)[2] or low-resolution-MS data.[3] Whereas not data indicated the kind of sugar linkage in these studies, OPC-*C*-hexosides up to DP2 have been described for *Crataegus*.[4] In the present LC-MS analysis of WS®1442 and its fractions, OPC hexosides DP1–5 were detected on the basis of their exact masses. In addition, a product ion spectrum was obtained of the OPC hexoside DP2 (Figure S5).

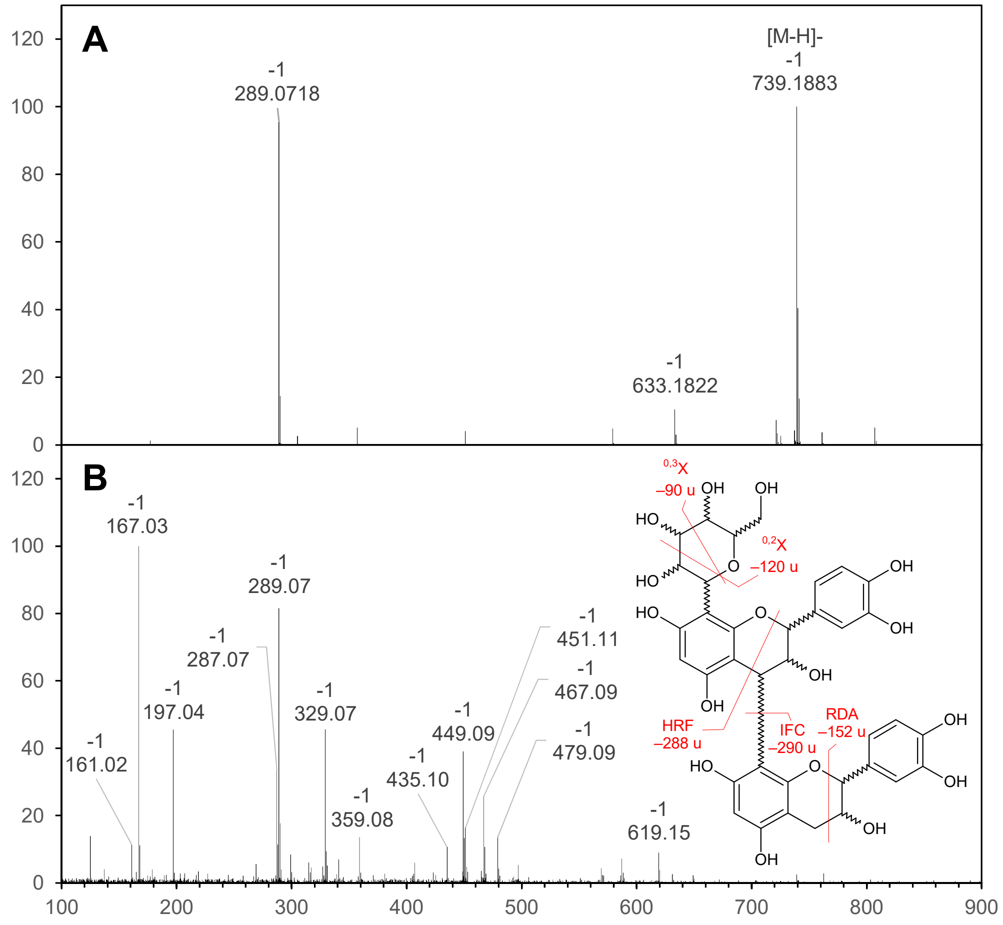

Figure S5. A) ESI mass spectrum of OPC hexoside DP2. The signal at *m/z* 289 is for the most part caused by a larger neighboring peak of a flavan-3-ol. B) Product ion spectrum of the deprotonated molecule (*m/z* 739, 40 eV).

The precursor ion at *m/z* 739.1883 $[C_{36}H_{35}O_{17}]^-$ (–0.4 mDa, mΣ 12.3) gave product ions that can in part be explained by the usual retro-Diels-Alder reaction (RDA), heterocyclic ring fission (HRF), and interflavan cleavage (IFC) as described for regular proanthocyanidins.[5] In addition, several signals indicate losses of 90 u and 120 u as observable with *C*-glycosidic flavonoids (^{0,3}X and ^{0,2}X, respectively).[6]

In total, 23 product ions of the deprotonated molecule at *m/z* 739 could be assigned to products of a combination of these five fragmentation reactions and eventual loss of water or carbon monoxide (Table S4). Product ions of specific diagnostic value are *m/z* 167 and 197 which result from HRF through the upper unit combined with ^{0,3}X or ^{0,2}X, respectively and indicate the presence of a *C*-hexosidic moiety at the A ring in position 6 or 8 of the upper unit (Figure 2B). OPC *C*-glycosides have also been described to occur in rhubarb,[7] *Cinnamomum spp.*[8] and –as a series of homologues up to DP6 on the basis of ESI-MS2-data– in *Hancornia speciosa*.[9]

Table S4. Assigned products ion of the *C*-glycosyl procyanidin dimer (OPC hexoside DP2).

m/z	intensity	formula	error / mDa	mΣ	fragmentation reactions
161.0240	75	$C_9H_5O_3$	4.0	55.8	M–120, IF, RDA, $-H_2O$
167.0345	660	$C_8H_7O_4$	0.5	13.4	M–120, HRF
179.0323	27	$C_9H_7O_4$	–3.7	95.2	M–120, IF, RDA
197.0443	300	$C_9H_9O_5$	1.3	20.4	M–90, HRF
[1]287.0646	221	$C_{12}H_{15}O_8$<br>$C_{15}H_{11}O_6$	–12.6<br>8.5	658.1<br>698.8	HRF<br>2 × HRF
289.0719	539	$C_{15}H_{13}O_6$	–0.1	32.1	IF
299.0687	56	$C_{16}H_{11}O_6$	12.6	n.a.	HRF, RDA
329.0650	301	$C_{17}H_{13}O_7$	1.7	42.1	M–120, IF
341.0598	47	$C_{18}H_{13}O_7$	–6.9	26.9	M–90, IF, $-H_2O$
359.0818	258	$C_{18}H_{15}O_8$	4.1	20	M–90, IF
407.1083	40	$C_{19}H_{19}O_{10}$	9.9	n.a.	2 × RDA, –CO
423.1206	20	$C_{19}H_{19}O_{11}$	27.4	363.9	HRF, RDA
435.0983	70	$C_{20}H_{19}O_9$	–5.0	116	2 × RDA
449.0908	258	$C_{24}H_{17}O_9$	–3.0	196.1	M–120, RDA, $-H_2O$
451.1051	108	$C_{24}H_{19}O_9$	–1.6	77.4	HRF
467.0929	170	$C_{24}H_{19}O_{10}$	–5.4	79.7	M–120, RDA
479.0920	36	$C_{25}H_{19}O_{10}$	6.4	13.3	M–90, RDA, $-H_2O$
497.1026	36	$C_{25}H_{21}O_{11}$	–6.4	88.8	M–90, RDA
569.1245	29	$C_{28}H_{25}O_{13}$	5.6	127.4	RDA, $-H_2O$
587.1398	48	$C_{28}H_{27}O_{14}$	–0.9	61.6	RDA
619.1493	49	$C_{32}H_{27}O_{13}$	3.6	55.8	M–120 [^{0,2}X]
649.1345	16	$C_{33}H_{29}O_{14}$	–21.7	n.a.	M–90 [^{0,3}X]
739.2040	17	$C_{36}H_{35}O_{17}$	16	65.4	–

[1] We suggest that the comparably large mass deviations for the suggested formulas result from the unresolved signals of two isobaric fragments.

3) Transcriptome analyses

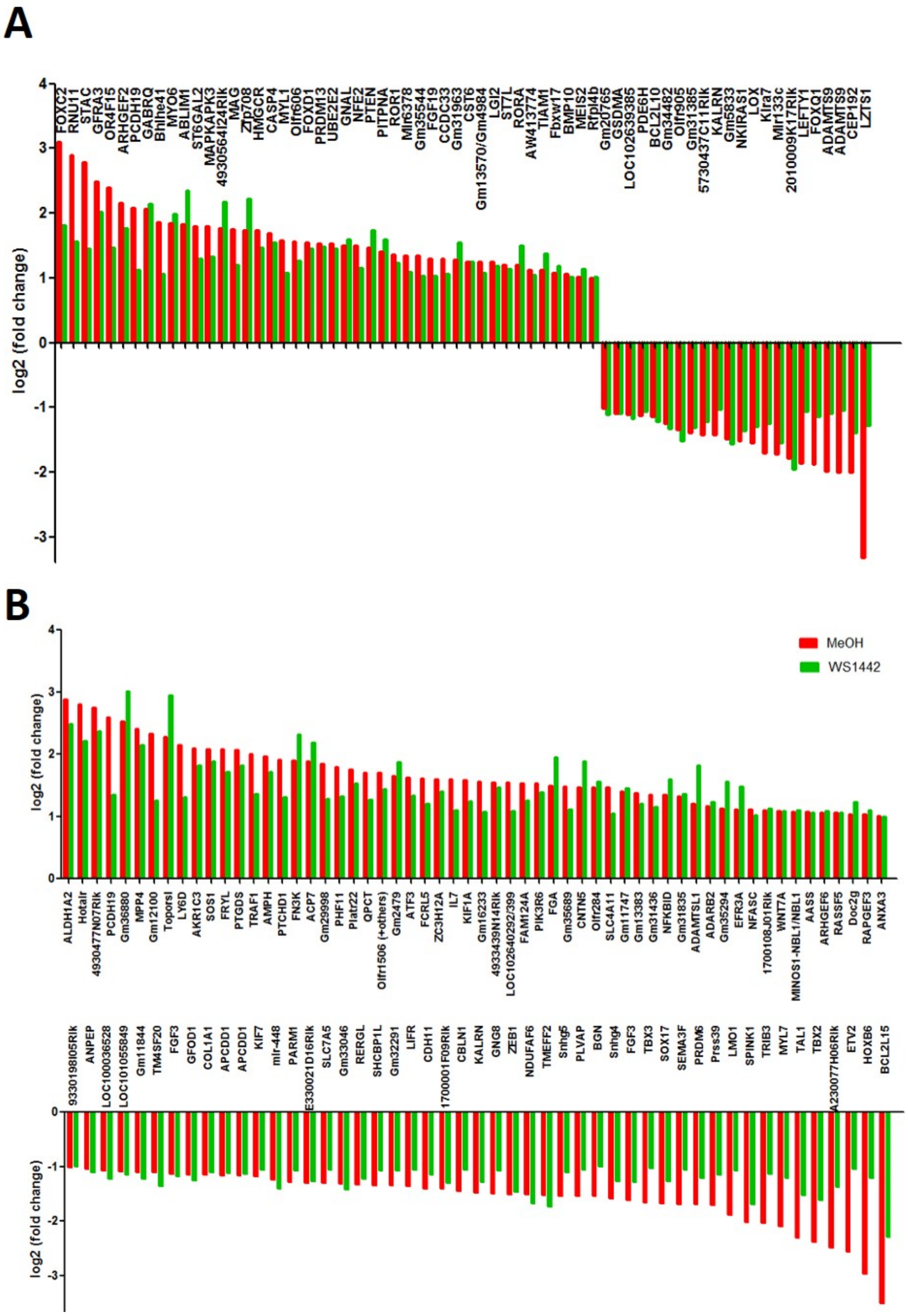

Figure S6. Log2(FC)-plot of all commonly regulated genes upon WS®1442 and MeOH eluate treatments during mESC differentiation. A) 66 commonly regulated and assigned genes after 6h treatments, B) 107 commonly regulated and assigned genes after 24h treatments.

4) Supplementary videos

Videos were recorded on an Eclipse TE200 (Nikon) inverted phase contrast microscope (10x objective).

Video S1: Beating cardiomyocytes on day 11 after WS®1442 (0.3 mg/mL) treatment (d4-6).

Video S2: Beating cardiomyocytes on day 11 after MeOH eluate (0.1 mg/mL) treatment (d4-6).

5) References

[1] S. Zumdick, F. Petereit, H. Luftmann, A. Hensel, *Pharmazie* **2009**, *64*, 286-288.

[2] a) J. Sendker, F. Petereit, M. Lautenschlager, N. Hellenbrand, A. Hensel, *Planta Med* **2013**, *79*, 45-51; b) J. Sendker, F. Petereit, M. Lautenschläger, N. Hellenbrand, A. Hensel, *Zeitschrift für Phytotherapie* **2013**, *34*.

[3] a) P. Liu, B. Yang, H. Kallio, *Food Chemistry* **2010**, *121*, 1188-1197; b) P. Liu, H. Kallio, D. Lu, C. Zhou, B. Yang, *Food Chem* **2011**, *127*, 1370-1377.

[4] M. G. E. Karar, N. Kuhnert, *Journal of Chemical Biology and Therapeutics* **2015**, *1*, 1000102.

[5] a) W. Friedrich, A. Eberhardt, R. Galensa, *European Food Research and Technology* **2000**, *211*, 56-64; b) L. Gu, M. A. Kelm, J. F. Hammerstone, Z. Zhang, G. Beecher, J. Holden, D. Haytowitz, R. L. Prior, *J Mass Spectrom* **2003**, *38*, 1272-1280.

[6] P. Waridel, J. L. Wolfender, K. Ndjoko, K. R. Hobby, H. J. Major, K. Hostettmann, *J Chromatogr A* **2001**, *926*, 29-41.

[7] Y. Kashiwada, G. Nonaka, I. Nishioka, *Chem Pharm Bull (Tokyo)* **1986**, *34*, 4083-4091.

[8] S. Morimoto, G. Nonaka, I. Nishioka, *Chemical and Pharmaceutical Bulletin* **1986**, *34*, 643-649.

[9] C. M. Rodrigues, D. Rinaldo, L. C. dos Santos, P. Montoro, S. Piacente, C. Pizza, C. A. Hiruma-Lima, A. R. Brito, W. Vilegas, *Rapid Commun Mass Spectrom* **2007**, *21*, 1907-1914.
